# Fault Feature Extraction for RV Reducers Based on IWHO-VMD and Effective Mode Reconstruction

**DOI:** 10.3390/s26175497

**Published:** 2026-08-30

**Authors:** Yueping Wang, Guodong Xu, Youkun Li, Xiaolong Zhang, Deqi Zuo

**Affiliations:** College of Mechanical and Transportation Engineering, Southwest Forestry University, Kunming 650224, China; murphywyp@gmail.com (Y.W.);

**Keywords:** RV reducer, fault feature extraction, variational mode decomposition, improved wild horse optimizer, mode screening and reconstruction, envelope spectrum analysis

## Abstract

Fault feature extraction for rotate vector (RV) reducers is hindered by weak impulsive components masked by noise, empirical parameter selection in conventional variational mode decomposition (VMD), and mode redundancy. To address these issues, an improved wild horse optimizer-based variational mode decomposition (IWHO-VMD) framework with effective-mode reconstruction is proposed. A two-stage search strategy and a composite fitness function integrating squared envelope spectrum (SES) negentropy, reconstruction error, and an effective-mode penalty are used to optimize the VMD mode number and penalty factor. Fault-related intrinsic mode functions are then selected using SES negentropy and normalized energy ratio, followed by effective mode reconstruction and Hilbert envelope spectrum analysis. The method is validated on two self-built RV reducer datasets involving rolling-element wear and planetary gear tooth-surface wear, together with a public crankshaft wear dataset, and compared with several optimization methods. It achieves an average characteristic-frequency identification accuracy of 98.95% across the three datasets. On the comparative dataset, IWHO-VMD achieves the lowest fitness value and reduces the number of convergence iterations by 25.0–50.0%; with effective-mode reconstruction, its average identification accuracy reaches 99.03%. The proposed framework improves VMD parameter adaptivity and enhances fault-feature representation, providing a reliable basis for RV reducer condition monitoring.

## 1. Introduction

Rotate vector (RV) reducers are key transmission components in industrial robots, precision machine tools, and aerospace equipment owing to their high transmission accuracy, load-carrying capacity, torsional rigidity, and durability [1]. Under prolonged operation, however, cyclic loading, impact, inadequate lubrication, and material fatigue can induce pitting, wear, and cracking in bearings and gears. If not detected promptly, these faults can impair positioning accuracy, cause unplanned shutdowns, and compromise system safety and reliability [2]. Consequently, the reliable extraction of fault-related components from vibration signals is essential for condition monitoring and fault diagnosis of RV reducers.

### 1.1. Technical Challenges

Fault feature extraction is complicated by the multistage coupled transmission structure of RV reducers. Localized bearing or gear wear generates weak periodic impulses that can be obscured by background noise, transmission-induced modulation, and overlapping spectral components [3,4]. Variational mode decomposition (VMD) is effective for nonlinear and non-stationary signal analysis, but its performance depends strongly on the mode number *K* and penalty factor α. Inappropriate parameter settings may cause under- or over-decomposition, mode mixing, redundant modes, or loss of fault-related information. Moreover, the resulting intrinsic mode functions (IMFs) contribute unequally to fault diagnosis; therefore, noise-dominated and weakly relevant modes must be screened before signal reconstruction and envelope-spectrum analysis.

### 1.2. Research Gap and Motivation

Although adaptive VMD parameter optimization and fault-sensitive IMF selection have been extensively investigated, two limitations remain. First, many optimization methods rely on a single entropy or impulsiveness index and therefore do not jointly evaluate fault sensitivity, reconstruction fidelity, and effective-mode retention [5]. Second, VMD parameter optimization and post-decomposition mode selection are often designed using different criteria, so a parameter combination judged optimal during decomposition may not produce the most informative reconstructed signal. A coordinated framework that links parameter search, decomposition evaluation, and effective-mode reconstruction is therefore needed to extract weak fault features from RV reducer vibration signals.

### 1.3. Major Contributions

To address these limitations, this study proposes a VMD-based fault-feature extraction framework using an improved wild horse optimizer (IWHO) and effective-mode reconstruction. The main contributions are as follows:

(1) An adaptive VMD parameter-optimization scheme is developed in which the mode number *K* is determined through a two-stage coarse-to-fine search, while the penalty factor α is optimized by IWHO for each candidate *K*. The optimizer incorporates Tent-map chaotic initialization, refraction mirror learning, and a hybrid perturbation strategy combining the Golden Sine Algorithm and Moth-Flame Optimization (MFO) to enhance search diversity, convergence stability, and local exploitation.
(2) A composite fitness function is formulated by integrating squared envelope spectrum (SES) negentropy, reconstruction-error mapping, and an effective-mode penalty. The resulting objective jointly evaluates fault-related impulsiveness, reconstruction fidelity, and effective-mode retention, thereby avoiding reliance on a single decomposition index.
(3) An effective-mode screening and reconstruction method is established by combining SES negentropy, normalized energy ratio, and an adaptive root-mean-square (RMS) threshold. Because the screening criterion is also incorporated into the effective-mode penalty, parameter optimization and subsequent IMF selection are coordinated within the same framework, enabling noise-dominated and redundant modes to be suppressed while fault-related components are retained.

### 1.4. Paper Organization

The remainder of this paper is organized as follows. Section 2 reviews fault-feature extraction methods for RV reducers and related rotating machinery, with emphasis on VMD parameter optimization and effective-mode selection. Section 3 details the proposed framework, including IWHO-based parameter search, the composite fitness function, effective-mode screening and reconstruction, theoretical fault-frequency analysis, experimental datasets, and comparative settings. Section 4 presents the sensitivity analyses, decomposition and reconstruction results, envelope-spectrum results, characteristic-frequency identification, and comparative evaluations. Section 5 discusses the underlying mechanisms, computational considerations, applicability, and limitations of the method. Section 6 summarizes the main findings and outlines directions for further validation.

## 2. Related Work

To position the proposed framework, this section reviews fault-feature extraction methods for RV reducers and related rotating machinery, with emphasis on adaptive VMD parameter optimization and fault-sensitive mode selection.

### 2.1. Fault Feature Extraction for RV Reducers and Related Rotating Machinery

Fault diagnosis of RV reducers has been investigated using both vibration and motor-current signals. Zhang et al. [6] applied VMD to the torsional-vibration analysis of an RV reducer and showed that it could mitigate frequency aliasing and interference. Xu et al. [7] combined information entropy with VMD for planetary gear fault analysis, further demonstrating the applicability of VMD to vibration-based feature extraction in RV transmission components. Raouf et al. [8] used motor-current features together with machine-learning methods to distinguish different health states of RV reducers in industrial robots. Although this approach broadened the available sensing modalities for RV reducer condition monitoring, it focused primarily on health-state classification rather than on the enhancement and interpretation of weak impulsive and modulation components in vibration signals.

In addition to VMD-based approaches, synchrosqueezing transform (SST) and empirical wavelet transform (EWT) have also been investigated for non-stationary vibration signal analysis in rotating machinery. Li and Liang [9] used a generalized SST for variable-speed gearbox fault diagnosis, while Hu et al. [10] applied a high-order SST to planetary gearbox diagnosis, improving time-frequency concentration and component reconstruction. For adaptive signal decomposition, Zhang et al. [11] developed an adaptive and concise EWT-based feature extraction method for bearing fault diagnosis, improving spectral segmentation and the extraction of periodic impulsive components from complex vibration signals.

These studies show that SST is well suited to tracking time-varying frequency components, whereas EWT facilitates adaptive spectral segmentation. For the constant-speed RV reducer signals considered in this study, VMD is retained as the basic decomposition framework because it provides a controllable modal representation that can be integrated with parameter optimization, IMF screening, and signal reconstruction [12]. The subsequent challenge is therefore to improve the adaptability of VMD parameter selection while ensuring that the decomposed modes retained for reconstruction contain diagnostically useful information.

### 2.2. VMD Parameter Optimization and Effective Mode Selection

Because VMD is sensitive to the mode number *K* and penalty factor α, metaheuristic optimization algorithms have increasingly been used to reduce dependence on empirical parameter selection. Li et al. [13] developed a hybrid-strategy variant of the wild horse optimizer (WHO) to improve population diversity, convergence speed, and resistance to local optima. He et al. [14] subsequently applied IWHO to optimize *K* and α, with Tent chaotic mapping introduced to improve population initialization and signal-decomposition quality. However, their optimization objective relied only on minimum envelope entropy, which may be insufficient to evaluate fault-related impulsiveness, modulation information, and reconstruction fidelity simultaneously. Guo et al. [15] proposed a CEEMDAN-CPO-VMD method for RV reducer fault diagnosis, reducing dependence on manually selected parameters but increasing computational complexity because of the dual-decomposition procedure. Qiu et al. [16] developed a WSO-VMD method for RV reducer diagnosis under variable-speed conditions and achieved effective signal denoising and fault-feature extraction.

Parameter optimization alone does not ensure that all decomposed IMFs are diagnostically useful. Noise-dominated, weakly relevant, or redundant modes may obscure characteristic frequencies if they are directly included in the reconstructed signal. Du et al. [17] optimized VMD parameters using the sparrow search algorithm and then selected IMFs through a multi-index criterion combining the Hoyer index and SES negentropy, thereby improving fault-feature extraction under complex noise. This study illustrates the value of quantitative mode screening after adaptive decomposition. Nevertheless, in many existing methods, parameter optimization and post-decomposition IMF selection remain sequential but separately designed stages: the parameter combination is optimized according to one objective, whereas the final modes are retained according to another.

### 2.3. Positioning of the Proposed Method

Taken together, previous studies have demonstrated the effectiveness of adaptive decomposition, metaheuristic VMD parameter optimization, and quantitative IMF screening. The principal unresolved issue is not the absence of these individual techniques, but the lack of coordination among them. A decomposition selected using a single entropy or impulsiveness criterion may emphasize prominent fault responses without adequately considering reconstruction fidelity or the proportion of diagnostically useful modes. Conversely, post hoc IMF selection cannot fully compensate for an unsuitable decomposition obtained during the preceding parameter-optimization stage. Multi-stage decomposition methods may alleviate some of these limitations, but often at the cost of additional computational complexity.

Against this background, the proposed method is positioned as a coordinated VMD-based fault-feature extraction framework rather than a simple combination of independently designed processing stages. The mode number *K* is determined through a two-stage coarse-to-fine search, while IWHO is used to optimize the penalty factor α for each candidate *K*. A composite fitness function integrates SES negentropy, reconstruction-error mapping, and an effective-mode penalty to evaluate fault sensitivity, reconstruction fidelity, and effective-mode retention. The resulting IMFs are then screened using SES negentropy, normalized energy ratio, and an adaptive root-mean-square threshold. Because the effective-mode criterion also contributes to the fitness penalty, parameter optimization and subsequent mode reconstruction are linked through a consistent evaluation mechanism.

## 3. Materials and Methods

This section presents the proposed IWHO-VMD framework, including parameter search, composite fitness evaluation, effective-mode reconstruction, fault-frequency analysis, experimental datasets, and comparative settings.

### 3.1. IWHO-Based VMD Parameter Optimization

VMD is an adaptive signal decomposition method that decomposes a nonlinear and non-stationary vibration signal into *K* IMFs with different center frequencies [18]. For an original vibration signal $x\left(t\right)$, the decomposed modes are constrained such that their sum reconstructs the original signal. The constrained variational model of VMD can be expressed as:

(1) $$\underset{{\{u}_{k}\},\{\omega_{k}\}}{min}\;\left\{\sum_{k=1}^{K}\;{∥\partial_{t}\left[\left(\delta \left(t\right)+\frac{j}{\pi t}\right)*u_{k}\left(t\right)\right]e^{-j\omega_{k}t}∥}_{2}^{2}\right\}$$

(2) $$s.t.\sum_{k=1}^{K}\;u_{k}\left(t\right)=x\left(t\right)$$

where *u_k_*(*t*) denotes the *k*-th IMF, *ω_k_* is its center frequency, *K* is the number of modes, *δ*(*t*) is the Dirac delta function, ∗ denotes the convolution operation, and *j* is the imaginary unit.

The decomposition performance of VMD depends strongly on the mode number *K* and penalty factor *α*. Improper parameter selection may cause mode mixing, redundant components, or poor reconstruction accuracy. Therefore, this study combines IWHO with a two-stage *K*-search strategy for VMD parameter optimization [19]. To overcome the uneven initialization, insufficient diversity, and premature convergence of the original WHO, Tent chaotic initialization, refraction mirror learning, and a hybrid perturbation strategy combining the Golden Sine Algorithm and MFO are introduced. These strategies improve population diversity, search stability, and local exploitation, enabling IWHO to provide a more reliable adaptive mechanism for VMD parameter selection.

First, Tent chaotic mapping is used to initialize the population. Compared with random initialization, Tent chaotic mapping improves the ergodicity of the initial population and helps individuals cover the search space more uniformly [20,21]. The initial position of the *p*-th individual in the *d*-th dimension is defined as:

(3) $$x_{p,d}^{\left(0\right)}=lb_{d}+c_{p,d}\left(ub_{d}-lb_{d}\right)$$

where $x_{p,d}^{\left(0\right)}$ is the initial position, $lb_{d}$ and $ub_{d}$ are the lower and upper bounds of the *d*-th dimension, and c*_p_*_,*d*_ ∈ (0, 1) is the chaotic variable generated by the Tent map.

Second, a refraction mirror learning strategy is introduced to increase population diversity and reduce the probability of search stagnation [22,23]. The new candidate position is generated according to the relationship between the current individual and the center of the search interval:

(4) $$x_{p,d}^{new}=o_{d}+\eta \left(o_{d}-x_{p,d}\right),o_{d}=\frac{lb_{d}+ub_{d}}{2}$$

where $x_{p,d}$ is the current position, $o_{d}$ is the center of the d-th search interval, and η is the refraction coefficient.

Finally, a hybrid perturbation strategy combining the Golden Sine Algorithm and MFO is introduced into the stallion position update process to enhance local exploitation [24]. The updated position is expressed as:

(5) $$P_{p}^{\left(g+1\right)}=\left\{\begin{array}{c} P_{p}^{\left(g\right)}|\sin\left(\rho_{1}\right)|-\rho_{2}\sin\left(\rho_{1}\right)|\rho_{3}W-\rho_{4}P_{p}^{\left(g\right)}|,R\leq 0.5\\ |W-P_{p}^{\left(g\right)}|e^{bl}\cos\left(2\pi l\right)+W,\;\;\;\;\;\;\;\;\;\;\;\;\;\;\;\;R>0.5 \end{array}\right.$$

where $P_{p}^{\left(g\right)}$ and $P_{p}^{\left(g+1\right)}$ denote the position of the *p*-th stallion at the *g*-th and (g + 1)-th iterations; *W* denotes the current best position found by the population; *ρ*_1_, *ρ*_2_, *ρ*_3_, *ρ*_4_, and *R* are random numbers in [0, 1]; *b* is the spiral shape parameter; and ℓ is the MFO spiral perturbation parameter.

Together, these three strategies enhance the search process within the two-stage parameter-selection framework. The composite fitness function used to evaluate each candidate (K, α) combination is defined in the following section.

### 3.2. Composite Fitness Function for VMD Parameter Optimization

Adaptive VMD parameter optimization requires a fitness function that comprehensively evaluates decomposition quality. Since a single index cannot simultaneously reflect impulsive feature preservation, reconstruction accuracy, and effective mode retention, this study integrates SES negentropy, reconstruction-error mapping, and an effective-mode penalty into a multi-index weighted fitness function to evaluate VMD under different parameter combinations and guide parameter search [25,26,27].

First, SES negentropy is introduced to evaluate the impulsive-feature representation ability of each IMF [28]. For the *k*-th IMF $u_{k}\left(t\right)$, the normalized squared envelope spectrum probability is calculated as:

(6) $$p_{k,m}=\frac{{\left|F\left\{{\left|u_{k}\left(t\right)+jH\left[u_{k}\left(t\right)\right]\right|}^{2}\right\}\left(v_{m}\right)\right|}^{2}}{\sum_{q=1}^{M}\;{\left|F\left\{{\left|u_{k}\left(t\right)+jH\left[u_{k}\left(t\right)\right]\right|}^{2}\right\}\left(v_{q}\right)\right|}^{2}}$$

where $p_{k,m}$ denotes the normalized squared envelope spectrum probability of the *k*-th IMF at the frequency bin $v_{m}$; $H\left(\cdot \right)$ denotes the Hilbert transform; $F\left\{\cdot \right\}$ denotes the Fourier transform; *j* is the imaginary unit; $v_{m}$ and $v_{q}$ are the *m*-th and *q*-th frequency bins, respectively; and *M* is the total number of frequency bins. Accordingly, the SES negentropy of the *k*-th IMF is defined as:

(7) $$S_{k}^{ses}=1+\frac{\sum_{m=1}^{M}\;p_{k,m}\ln p_{k,m}}{\ln M}$$

The top *M_t_* modes with the highest scores are selected to calculate the average impulsive representation index as:

(8) $${\bar{S}}_{ses}=\frac{1}{M_{t}}\sum_{r=1}^{M_{t}}\;S_{\left(r\right)}^{ses}$$

where $S_{\left(r\right)}^{\mathrm{ses}}$ represents the *r*-th largest SES negentropy after sorting in descending order, and *M_t_* is the number of selected high-negentropy modes.

Second, to evaluate the ability of VMD to reconstruct the original signal, the reconstruction error index is introduced [29]. Let the reconstructed signal be:

(9) $$\hat{x}\left(t\right)=\sum_{k=1}^{K}\;u_{k}\left(t\right)$$

The reconstruction error is then defined as:

(10) $$E_{rec}=\frac{∥x-\hat{x}{∥}_{2}}{∥x{∥}_{2}+\varepsilon }$$

where ε is a small positive constant introduced to avoid division by zero. Since *E_rec_* is usually numerically small and differs in scale from the other evaluation indices, an exponential mapping is adopted to convert it into a dimensionless penalty term:

(11) $$E_{score}=1-\exp\left(-\frac{E_{rec}}{E_{ref}}\right)$$

where *E_ref_* is a reference scale parameter.

Third, an effective-mode penalty is introduced to evaluate the retention of fault-related impulsive modes [30]. Let *E_ffc_* denote the number of IMFs satisfying the effective-mode criterion defined in Section 3.3, and *K* denote the total number of decomposed modes. The effective-mode penalty is defined as:

(12) $$R_{eff}=1-\frac{E_{ff_{c}}}{K}$$

where *R_eff_* is used as a minimization-oriented penalty term. A smaller *R_eff_* indicates a higher proportion of effective fault-related IMFs in the decomposition result.

In summary, the composite fitness function constructed in this study is formulated as:

(13) $$F\left(K,\alpha \right)=w_{1}\left(1-{\bar{S}}_{ses}\right)+w_{2}E_{score}+w_{3}R_{eff}$$

where $w_{1}$, $w_{2}$ and $w_{3}$ are non-negative weighting coefficients satisfying $w_{1}+w_{2}+w_{3}=1$.

The three terms in the composite fitness function serve complementary roles. SES negentropy is used as the primary fault-sensitive term because localized bearing and gear faults generally produce repetitive impulsive and cyclostationary responses that are reflected in the squared envelope spectrum. The reconstruction-error term acts as a fidelity constraint, preventing the optimization from favoring highly impulsive decompositions at the expense of signal reconstruction. The effective-mode penalty further links parameter optimization to the subsequent mode-screening stage by penalizing decompositions in which only a small proportion of the modes are retained for fault-related reconstruction. The resulting fitness function therefore jointly evaluates fault sensitivity, reconstruction fidelity, and effective-mode retention.

Based on these complementary roles, SES negentropy was assigned the largest weight, while the reconstruction-error term and effective-mode penalty were used as auxiliary constraints. Subject to $w_{1}+w_{2}+w_{3}=1$, six representative weight combinations were evaluated through the local sensitivity analysis described in Section 4.1. The final weights were set to $\;w_{1}$ = 0.45, $\;w_{2}$ = 0.30, and $\;w_{3}$ = 0.25, and were fixed for all subsequent experiments.

### 3.3. Effective Mode Selection and Signal Reconstruction

The IWHO-VMD procedure yields *K* IMFs with different spectral and energy characteristics. Because some modes may be dominated by background noise, weak disturbances, or redundant information, a quantitative screening criterion is required to identify the modes retained for signal reconstruction [17,31].

To jointly consider impulsive-feature representation and energy contribution, the normalized energy ratio of the *k*-th IMF is defined as:

(14) $$r_{k}=\frac{\sum_{n=1}^{N}\;u_{k}^{2}(n)}{\sum_{q=1}^{K}\;\sum_{n=1}^{N}\;u_{q}^{2}(n)},{\bar{r}}_{k}=\frac{r_{k}}{\underset{1\leq q\leq K}{max}\;r_{q}}$$

where $u_{k}\left(n\right)$ denotes the *n*-th sampling point of the *k*-th IMF, *N* is the number of sampling points, $r_{k}$ is the energy ratio of the *k*-th IMF, and ${\bar{r}}_{k}$ is the normalized energy ratio.

The comprehensive evaluation index of the *k*-th IMF is then constructed by combining SES negentropy and normalized energy ratio:

(15) $$C_{k}=\lambda S_{k}^{\mathrm{ses}}+\left(1-\lambda \right){\bar{r}}_{k}$$

where λ ∈ [0, 1] controls the relative contributions of SES negentropy and normalized modal energy. A sensitivity analysis was conducted on the RV_Reducer@PG dataset using λ = 0.65, 0.70, 0.75, 0.80, and 0.85 under otherwise identical settings. Based on the overall fault-feature extraction and mode-independence performance, λ = 0.75 was adopted. The adaptive threshold for effective mode selection is defined as:

(16) $$T_{C}=\sqrt{\frac{1}{K}\sum_{i=1}^{K}\;C_{i}^{2}}$$

IMFs satisfying $C_{k}\geq T_{C}$ are retained to form the effective mode set Ω, whereas the remaining modes are discarded. The reconstructed signal is then obtained as:

(17) $$x_{r}\left(t\right)=\sum_{k\in \Omega }\;u_{k}\left(t\right)$$

Unlike impulsiveness-only screening, the proposed criterion combines SES negentropy and normalized energy ratio to evaluate fault-related impulsiveness and modal contribution, with greater weight assigned to SES negentropy. The RMS threshold adaptively determines the retained IMFs. It is effective when fault-related components are concentrated in a few modes and remain distinguishable from noise, but its reliability may decrease under strong noise because overlapping modal scores can hinder the separation of weak fault modes. The reconstructed signal suppresses noise-dominated and redundant components while preserving fault-related impulsive and modulation information, providing a basis for the theoretical frequency analysis in the following section.

### 3.4. RV Reducer Structure and Fault Characteristic Analysis

**Figure 1:**
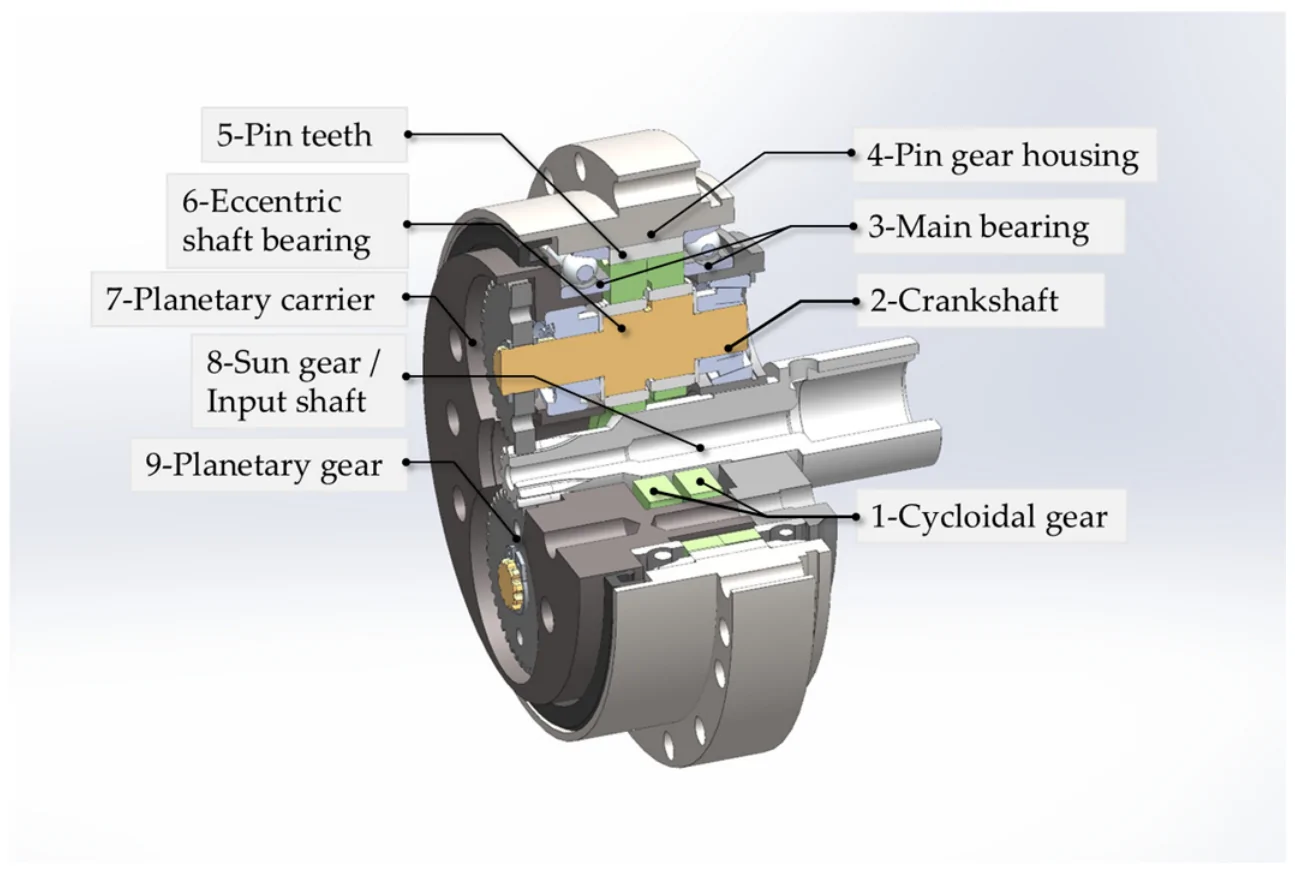
Three-dimensional structure and main components of the RV reducer.

An RV reducer is a high-precision enclosed differential transmission mechanism composed of a first-stage planetary gear reducer and a second-stage cycloidal pinwheel reducer in series. As shown in Figure 1, power is transmitted from the input shaft to the sun gear, planetary gears, crankshafts, and dual cycloidal gears, achieving two-stage speed reduction and torque amplification. Finally, the motion is delivered to the output disc through the crankshafts and support bearings, enabling low-speed and high-torque output [32,33].

The overall transmission ratio of an RV reducer is determined by its two reduction stages. The 180° phase arrangement of the dual cycloidal gears reduces radial inertial forces and vibration shocks, improving accuracy and stability. However, the multi-stage coupled structure also increases signal complexity and strengthens modulation phenomena.

For the two self-built datasets considered in this study, the investigated fault components are rolling-element wear of the output-end angular contact ball bearing and tooth-surface wear of the planetary gear [34]. For the output-end bearing, rolling-element defects generate periodic impacts when the damaged element repeatedly contacts the inner and outer raceways. Therefore, the envelope spectrum should consider not only the theoretical rolling-element fault frequency *f_BSF_*, but also its harmonics and modulation components caused by the multi-stage transmission of the RV reducer [32,35]. The observed fault-related frequency can be expressed as:

(18) $$f_{obs}=h\cdot f_{BSF}\pm l\cdot f_{m}$$

where *f_obs_* is the observed frequency, $f_{BSF}$ is the rolling-element fault characteristic frequency, and *f_m_* is the modulation frequency; *h* = 1,2,3,… is the harmonic order, and *l* = 0, 1, 2, … is the sideband order. In practice, the output-disc rotational frequency and crankshaft revolution frequency can be regarded as the main modulation frequencies. Thus, the common candidate components of rolling-element faults include:

(19) $$f_{BSF},\;2f_{BSF},f_{BSF}\;\pm \;f_{m},2f_{BSF}\;\pm \;f_{m},\cdots$$

For planetary gear tooth-surface wear, the fault features are mainly determined by the planetary gear self-rotation frequency *f*_3_ and the sun gear-planetary gear meshing frequency *f*_1*c*_ [36]. As the worn tooth periodically enters the meshing region, the vibration signal contains *f*_3_ [37] and its harmonics in the low-frequency range. Meanwhile, tooth-surface wear modulates the meshing vibration, producing sidebands around *f*_1*c*_ and its harmonics. Therefore, the typical components are:

(20) $$hf_{3},hf_{1c}\;\pm \;lf_{3}$$

For crankshaft wear, the crankshaft self-rotation frequency *f*_3_ and its harmonics are regarded as the principal candidate fault-related components in the subsequent analysis of the public dataset. These frequency relationships provide the theoretical basis for the subsequent envelope-spectrum-based identification of bearing, planetary gear, and crankshaft faults. The main geometric parameters and theoretical frequency expressions based on the RV20E structural parameters are listed in Table 1 and Table 2.

### 3.5. Experimental Platform and Datasets

**Figure 2:**
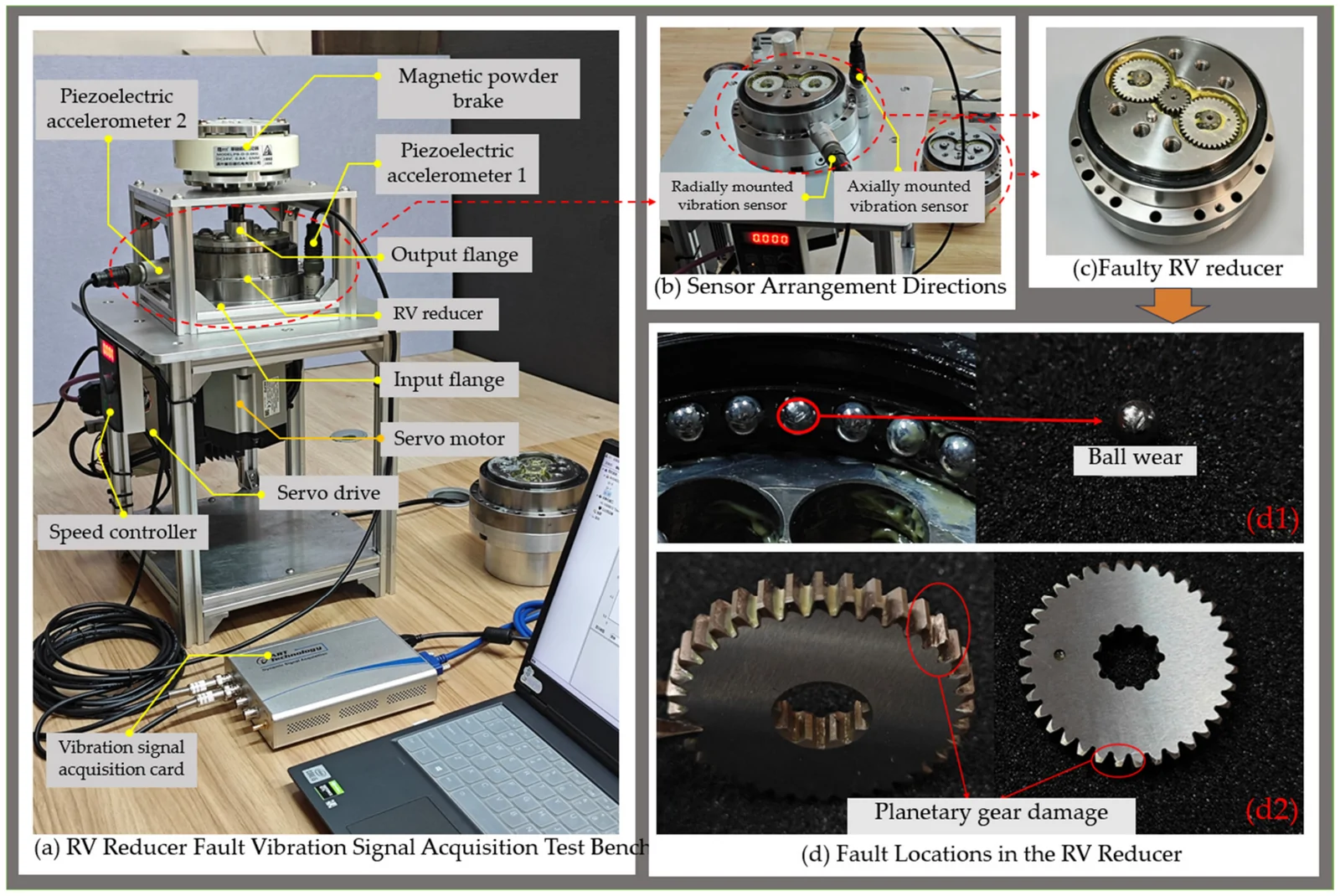
RV reducer fault locations and vibration-signal acquisition test bench: (**a**) test bench; (**b**) radial and axial sensor arrangement; (**c**) faulty RV reducer; (**d1**) rolling-element wear; and (**d2**) planetary gear tooth-surface wear.

Three fault datasets were used to evaluate the proposed method. Two self-built datasets, denoted as RV_Reducer@BB and RV_Reducer@PG, correspond to rolling-element wear of the output-end angular contact ball bearing and tooth-surface wear of the planetary gear, respectively. The rolling-element wear defect was approximately 2 mm in length and 1 mm in width, whereas the planetary gear wear defect extended across the tooth surface with a width of approximately 1.5 mm. The locations and morphologies of these artificially introduced faults are shown in Figure 2d.

**Figure 3:**
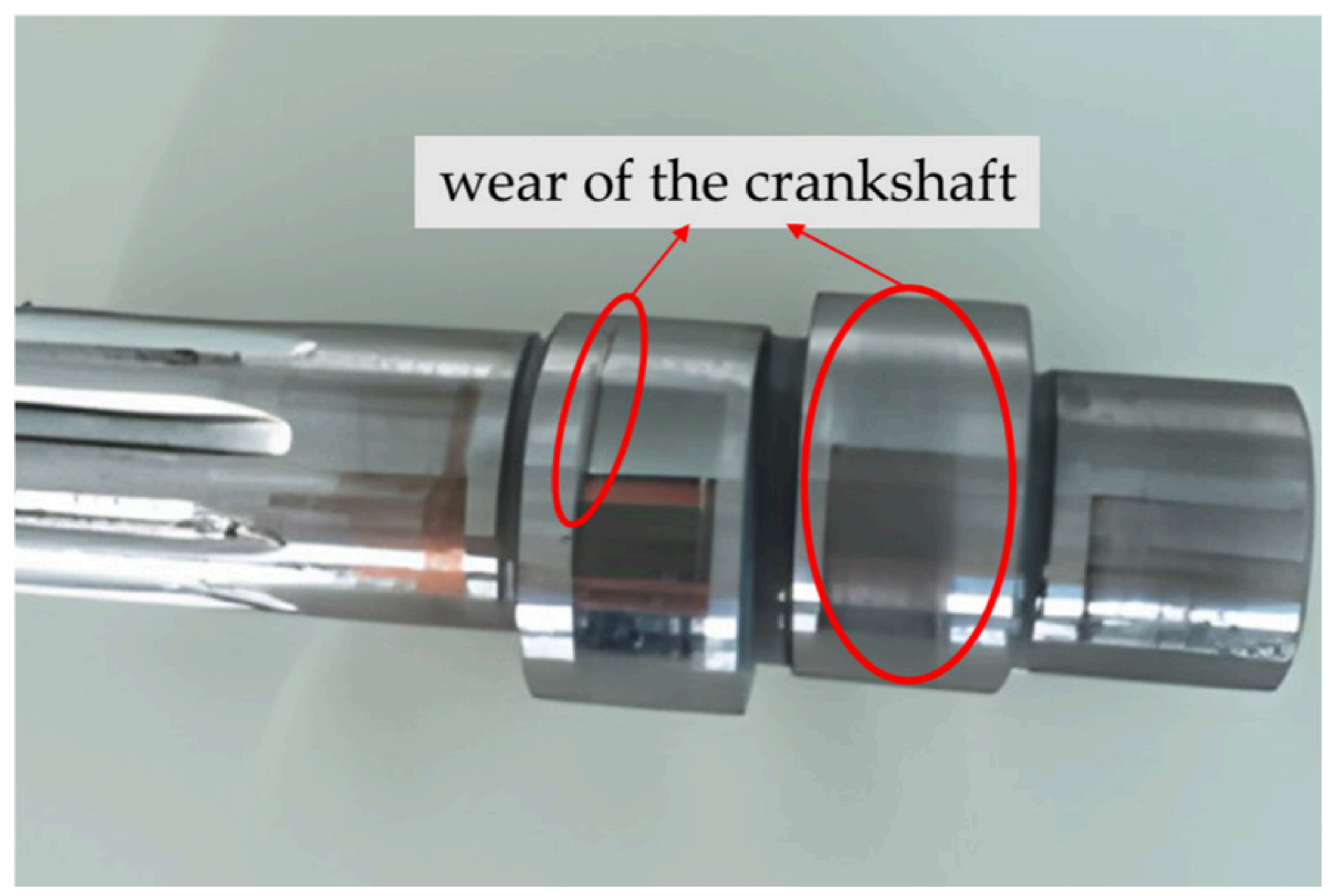
Crankshaft wear fault.

For supplementary validation, a public crankshaft wear dataset, denoted as RV_Reducer@CS, was also included. The dataset was provided by the Instrumentation Technology and Economy Institute and accessed through the National Basic Science Data Center [38]. The corresponding crankshaft wear morphology is shown in Figure 3.

The RV_Reducer@BB and RV_Reducer@PG datasets were acquired using the self-built vibration test platform shown in Figure 2. The test bench mainly consisted of a servo motor, servo drive, speed controller, RV reducer (Nabtesco Corporation, Tokyo, Japan), magnetic powder brake, accelerometers, and an ART-Technology USB8812 vibration data acquisition card (Beijing ART Technology Development Co., Ltd., Beijing, China). The RV reducer was connected to the servo motor and magnetic powder brake through flanges. During data acquisition, the magnetic powder brake applied a load torque of 12 N·m to the RV reducer, thereby providing controlled and consistent operating conditions for method evaluation.

Two XY126D100 accelerometers (Yangzhou Xiyuan Electronic Technology Co., Ltd., Yangzhou, China) with a measurement range of ±50 g were mounted by threaded fastening at the output end of the RV reducer. As shown in Figure 2b, one sensor was oriented radially and the other axially to record vibration responses in the two directions. The output-end position was selected because it is close to the investigated bearing and provides a relatively direct transmission path for vibrations generated by the internal components. Preliminary comparison of the two measured directions showed that the radial signal contained more prominent fault-related impulsive components; therefore, the radial vibration signal was used for the subsequent analysis.

For both the rolling-element wear and planetary gear tooth-surface wear conditions, the input rotational speed was maintained at 2500 r/min under the constant load torque of 12 N·m. The sampling frequency was set to 25.6 kHz, and the duration of each acquisition was 5 s, resulting in 1.28 × 10^5^ sampling points per signal. A total of 12 independent vibration recordings were collected for each fault condition, with an interval of 1 min between consecutive acquisitions. These experimental settings provided sufficient vibration samples for subsequent IWHO-VMD parameter optimization, effective mode selection, signal reconstruction, and envelope-spectrum analysis.

The two self-built fault conditions were artificially introduced to ensure repeatability and experimental control. Because prefabricated defects may generate more distinct vibration signatures than naturally developing incipient faults, the corresponding datasets are used primarily to evaluate fault-feature extraction under controlled conditions rather than to represent early-stage faults encountered in practice.

### 3.6. Fault Feature Extraction Procedure and Parameter Optimization Settings

**Figure 4:**
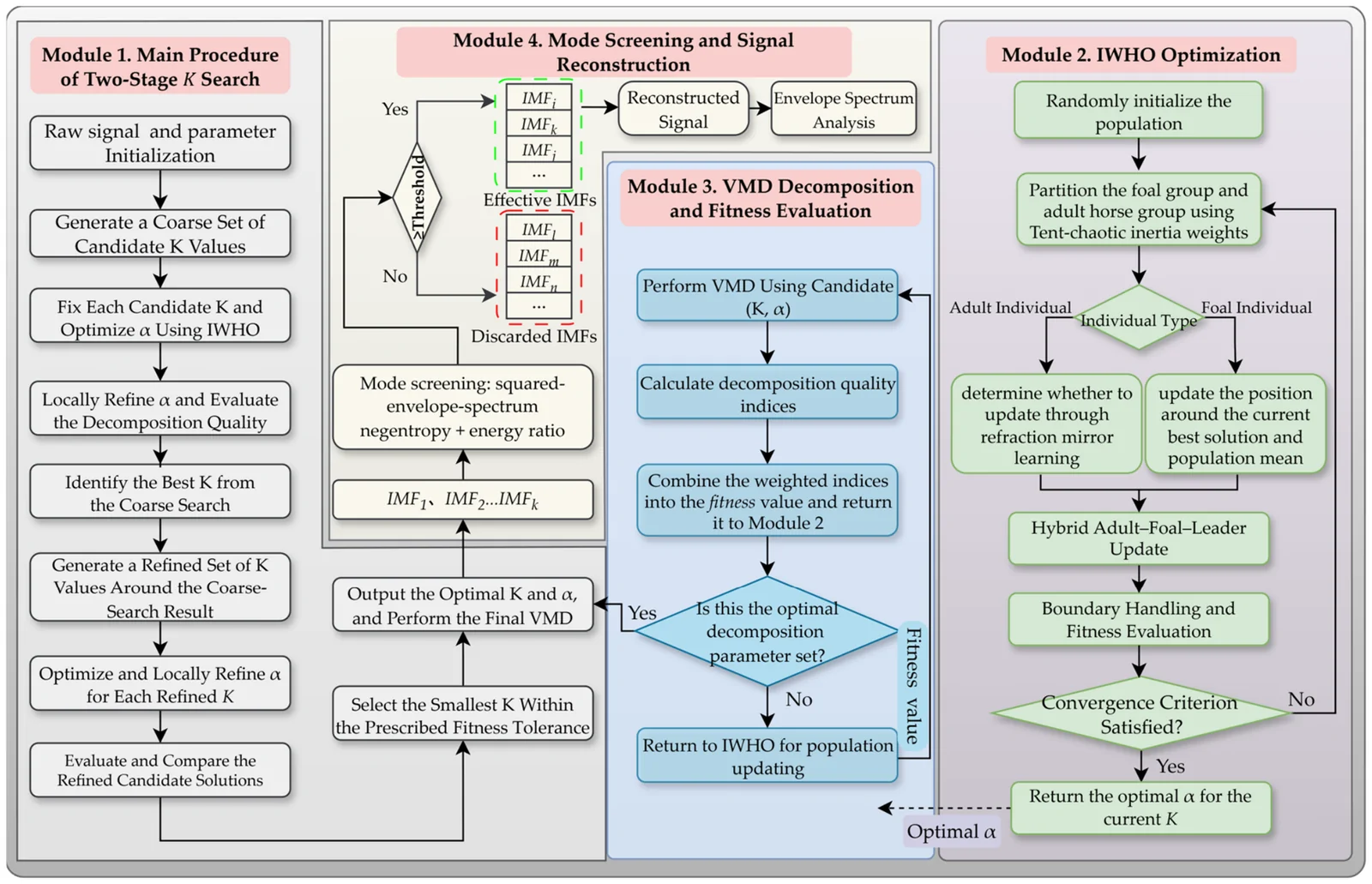
Flowchart of the proposed IWHO-VMD parameter optimization, mode screening, and signal reconstruction procedure.

Based on the datasets and acquisition settings described above, Figure 4 summarizes the overall IWHO-VMD fault-feature extraction workflow. The framework integrates adaptive parameter optimization, VMD, effective-mode screening, signal reconstruction, and envelope-spectrum analysis.

During VMD parameter optimization, the IWHO population size was set to 30. Preliminary searches were conducted over wider intervals of *K* and α to determine practical bounds for the formal experiments. An unsuitable value of *K* may result in under- or over-decomposition, mode mixing, or redundant modes, whereas an inappropriate value of α may cause spectral overlap or excessively restricted modal bandwidths. The preliminary results indicated that competitive solutions were concentrated mainly within *K* ∈ [4, 15] and α ∈ [800, 5000]. These intervals were therefore adopted to balance search coverage and computational efficiency. The consecutive stability-count threshold *S_t_* and convergence-stability threshold *S_h_* were set to 20 and 10^−5^, respectively.

To balance the stability and efficiency of *K*-value selection, a two-stage search strategy was adopted. In the first stage, *K* was coarsely sampled over the predefined range with a step size of 2. For each candidate *K*, IWHO was used to optimize the penalty factor α, followed by local grid refinement around *α_best_*. The refined fitness value was then used as the evaluation score for that candidate *K*.

In the second stage, the center mode number *K_center_*, identified during the first-stage screening, was used to construct a local refinement set within its neighborhood:

(21) $$K_{refine}=\left[K_{center}-2,K_{center}+2\right]$$

All integer values of *K* within this range were evaluated, while the search range of α was narrowed to improve local search accuracy and parameter-selection stability.

To reduce the risk of truncating a potential optimum at the initial search bounds, a boundary-triggered range-expansion mechanism was introduced. If the selected *K* reached either boundary, the interval was extended in the corresponding direction and the optimization was repeated. The same procedure was applied when the optimized α was located at or sufficiently close to a boundary. Boundary solutions were therefore treated as provisional candidates rather than being accepted directly as the final optimum.

### 3.7. Comparative Experiment Design and Evaluation Metrics

WHO-based VMD (WHO-VMD), particle swarm optimization-based VMD (PSO-VMD), whale optimization algorithm-based VMD (WOA-VMD), and grey wolf optimization-based VMD (GWO-VMD) were selected as baseline methods. All comparative experiments were conducted on the RV_Reducer@PG dataset so that parameter-search performance, decomposition quality, and fault-feature extraction could be evaluated under the same dataset and experimental conditions.

To ensure a controlled comparison, all methods used the same input signal, composite fitness function, VMD parameter ranges, population size, maximum number of iterations, convergence criteria, and subsequent mode-screening and reconstruction procedure. Only the parameter-optimization algorithm was varied. This design provides a consistent basis for comparing the search behavior of the different optimizers. Because some terms in the composite fitness function are also used to characterize decomposition quality, complementary metrics not directly included in the optimization objective were additionally considered. The unified experimental settings are listed in Table 3.

The comparison was conducted at three levels. Parameter-optimization performance was evaluated using the final best fitness value and the number of convergence iterations. Decomposition quality was characterized by the reconstruction error, effective-mode proportion, and mean inter-mode correlation [28]. Fault-feature extraction performance was assessed using the peak-to-noise ratio (PNR), fault peak ratio (FPR), number of identifiable fault features, and characteristic-frequency identification accuracy.

Because all compared optimization algorithms are stochastic, each method was independently run 10 times under identical settings. For each run, the final best fitness value, convergence iterations, PNR, FPR, number of identifiable fault features, and characteristic-frequency identification accuracy were recorded. The results were reported as mean ± standard deviation (SD). Pairwise differences between IWHO-VMD and each baseline method were assessed using two-sided permutation tests based on the Mann–Whitney *U* statistic. For each performance metric, the resulting four *p*-values were adjusted using the Holm procedure, and *p_adj_* < 0.05 was considered statistically significant.

The reconstruction error *E_rec_* is defined in Equation (10). Because the effective-mode penalty *R_eff_* defined in Equation (12) is a minimization-oriented quantity, the effective-mode proportion used for decomposition-quality comparison is defined as:

(22) $$P_{\mathrm{eff}}=\frac{Eff_{c}}{K}=1-R_{\mathrm{eff}}$$

where *E_ffc_* is the number of IMFs satisfying the effective-mode selection criterion and *K* is the total number of decomposed modes. A larger *P_eff_* indicates that a greater proportion of the decomposed modes contains fault-related information.

Following previous studies that used inter-mode dependence to evaluate decomposition quality [29], the mean inter-mode correlation is introduced as a complementary metric to quantify statistical dependence and information redundancy among the decomposed IMFs.

(23) $$M_{ic}=\frac{2}{K(K-1)}\sum_{i=1}^{K-1}\;\sum_{j=i+1}^{K}\;|\rho (u_{i},u_{j})|$$

where $\rho \left(u_{i},u_{j}\right)$ denotes the Pearson correlation coefficient between the *i*-th and *j*-th IMFs. A smaller *M_ic_* indicates weaker inter-mode correlation, lower information redundancy, and better mode independence.

It should be noted that *E_rec_* and *R_eff_* have already been incorporated into the composite fitness function. Therefore, the reconstruction error and effective-mode proportion are used mainly to characterize the resulting decomposition rather than as fully independent external validation metrics. In contrast, *M_ic_* is introduced as a complementary indicator that is not directly included in the fitness function.

The fault peak ratio (FPR) is calculated as the ratio of the spectral energy integrated within ±2 Hz of all theoretical fault-related frequencies to the total envelope-spectrum energy over 0–2000 Hz. Higher PNR and FPR values indicate greater fault-peak prominence and stronger concentration of fault-related spectral energy [39].

## 4. Results

This section evaluates the proposed framework in terms of parameter optimization, decomposition quality, effective-mode reconstruction, characteristic-frequency identification, and comparative performance.

### 4.1. Parameter Optimization and Sensitivity Analysis

**Figure 5:**
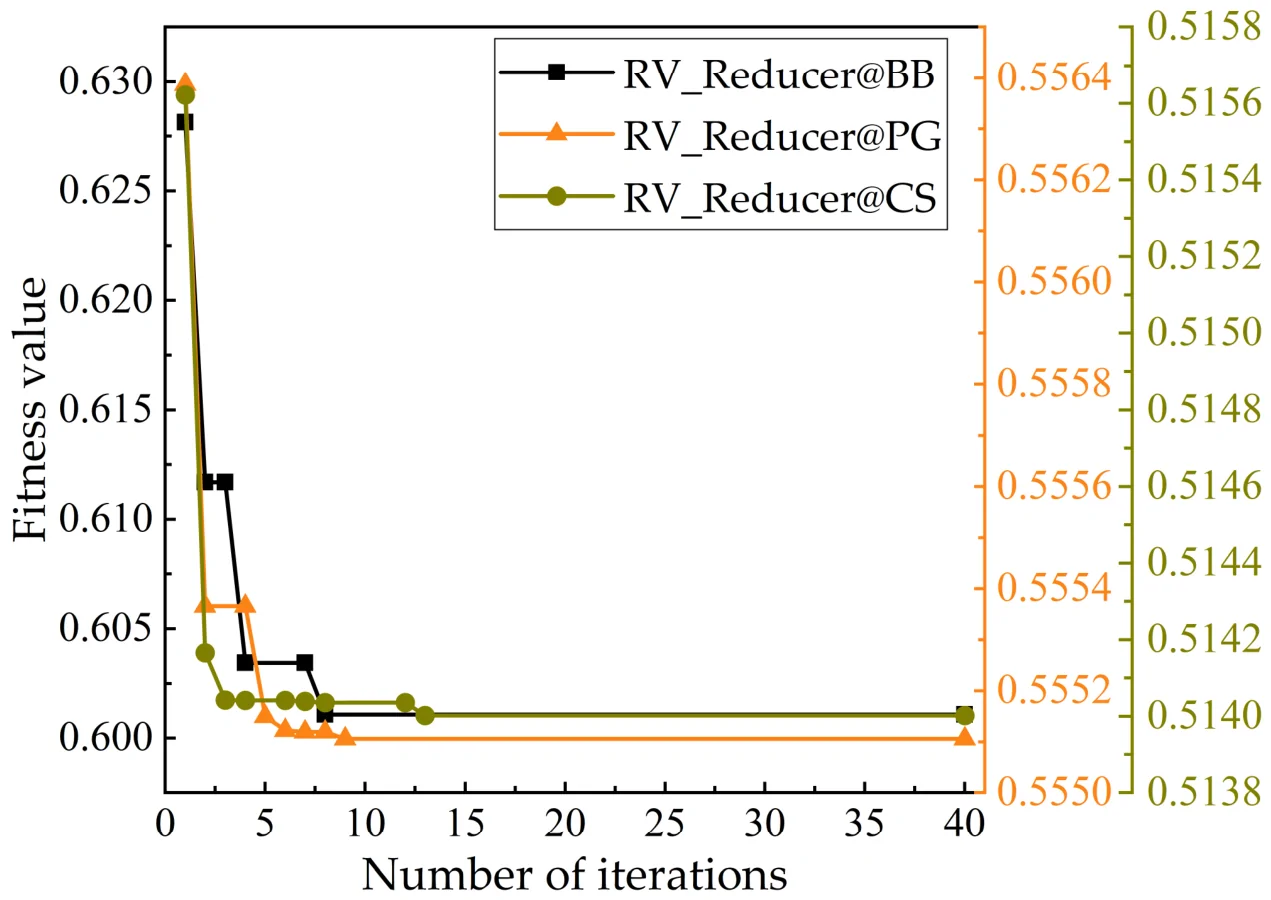
Convergence curves for the three RV reducer fault datasets.

The convergence behavior of IWHO during VMD parameter optimization was first evaluated on the three fault datasets by recording the evolution of the best fitness value over iterations, as shown in Figure 5.

For RV_Reducer@BB, RV_Reducer@PG, and RV_Reducer@CS, the fitness curves decrease rapidly during the early iterations and subsequently approach stable values, indicating consistent convergence behavior across the three fault conditions.

The resulting optimal VMD parameter combinations are *K* = 10 and α = 4247.18 for RV_Reducer@BB, *K* = 14 and α = 2188.13 for RV_Reducer@PG, and *K* = 5 and α = 1093.21 for RV_Reducer@CS. These optimized parameter combinations are subsequently used for VMD and effective mode analysis.

**Figure 6:**
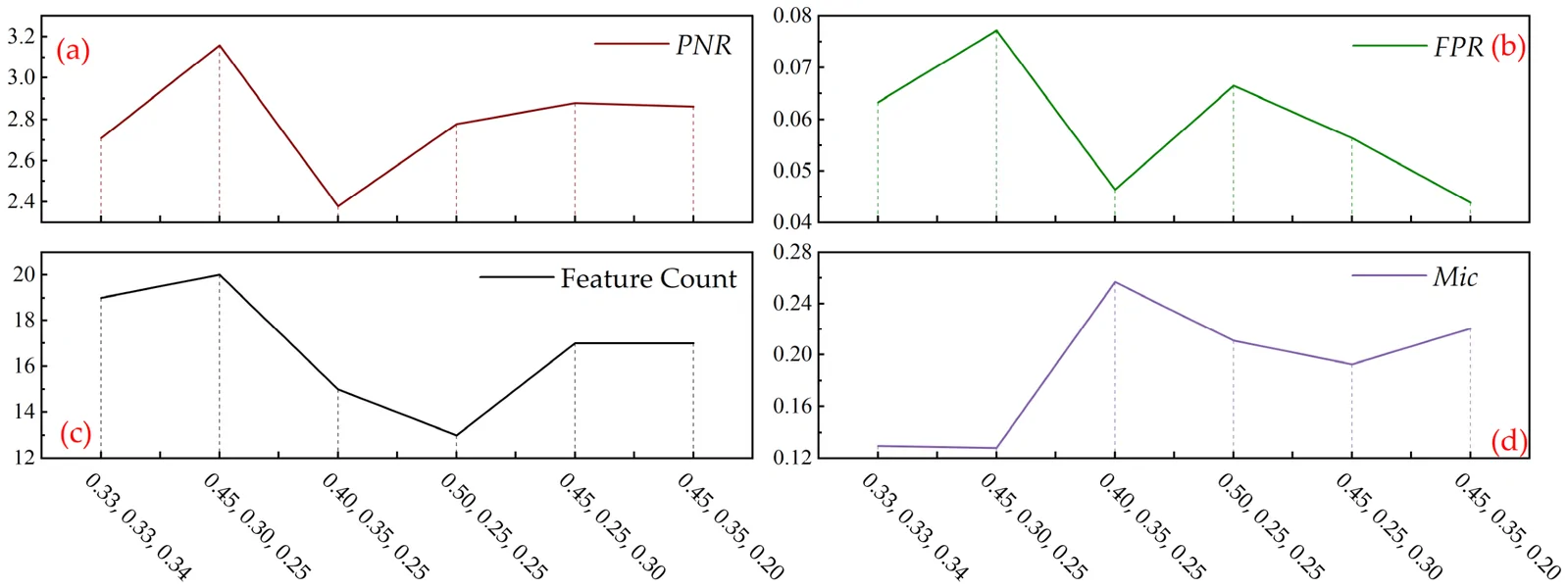
Sensitivity of the composite-fitness weights on the RV_Reducer@PG dataset: (**a**) peak-to-noise ratio (PNR); (**b**) fault peak ratio (FPR); (**c**) number of identifiable fault features; and (**d**) mean inter-mode correlation *M_ic_*. Each horizontal-axis label represents the weight vector (w_1_, w_2_, w_3_).

A local sensitivity analysis was then conducted to examine the influence of the composite-fitness weights. As shown in Figure 6, the adopted weight vector (0.45, 0.30, 0.25) yielded the highest PNR, FPR, and identifiable-feature count, together with the lowest mean inter-mode correlation among the six tested combinations. Because neighboring weight combinations did not improve all four indicators simultaneously, the selected weights represent a locally robust compromise within the investigated range rather than a theoretically unique or globally optimal solution.

The sensitivity of the effective-mode weighting coefficient λ was also evaluated at values of 0.65, 0.70, 0.75, 0.80, and 0.85. The setting λ = 0.75 yielded the highest PNR (3.1575), the largest identifiable-feature count (20), and the lowest mean inter-mode correlation (0.1281), while its FPR of 0.0772 was close to the maximum value of 0.0775 obtained at λ = 0.80. Accordingly, λ = 0.75 was selected as the best overall trade-off among the evaluated indicators.

### 4.2. VMD Results

**Figure 7:**
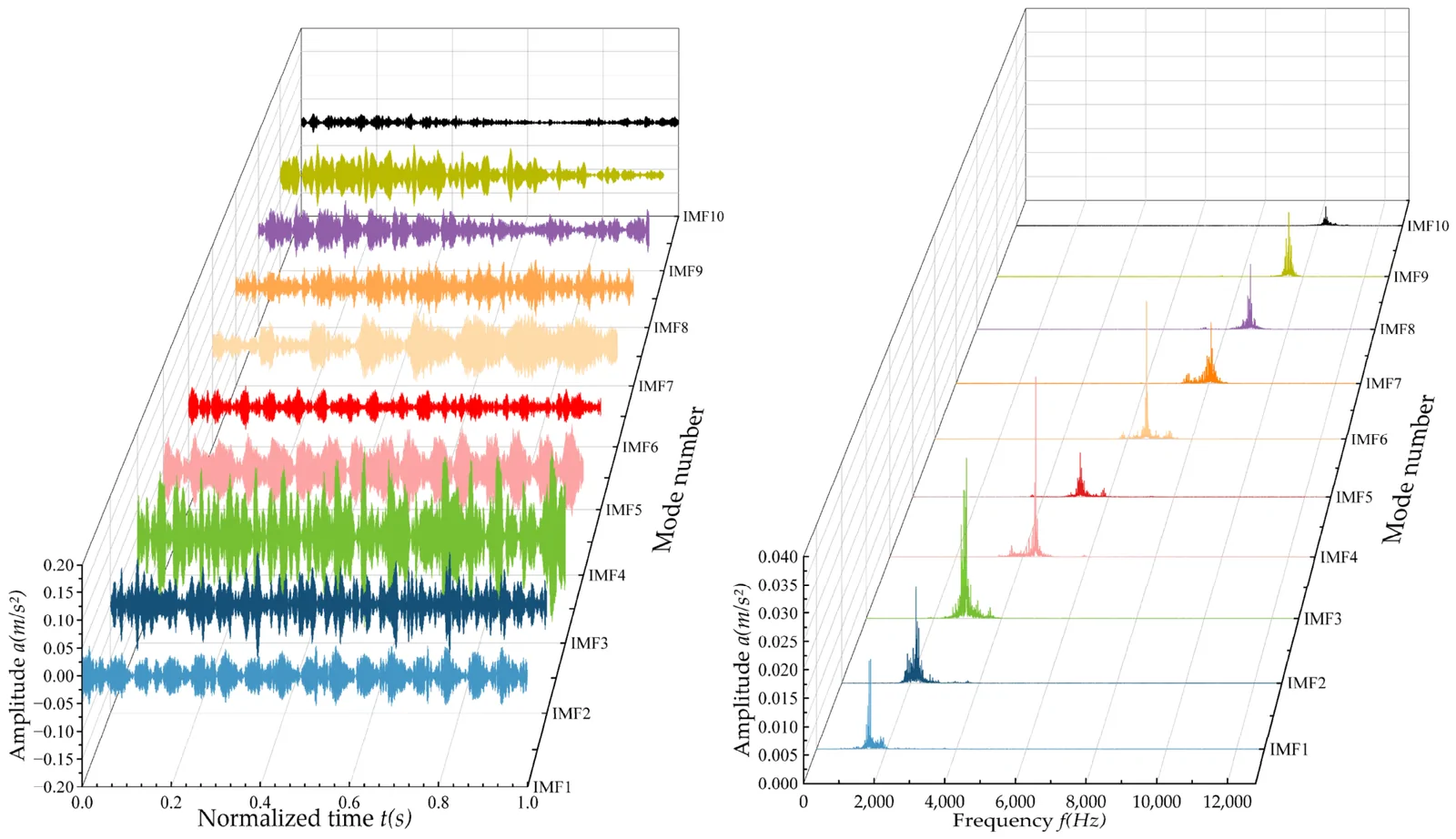
IWHO-VMD results for the RV_Reducer@BB dataset.

**Figure 8:**
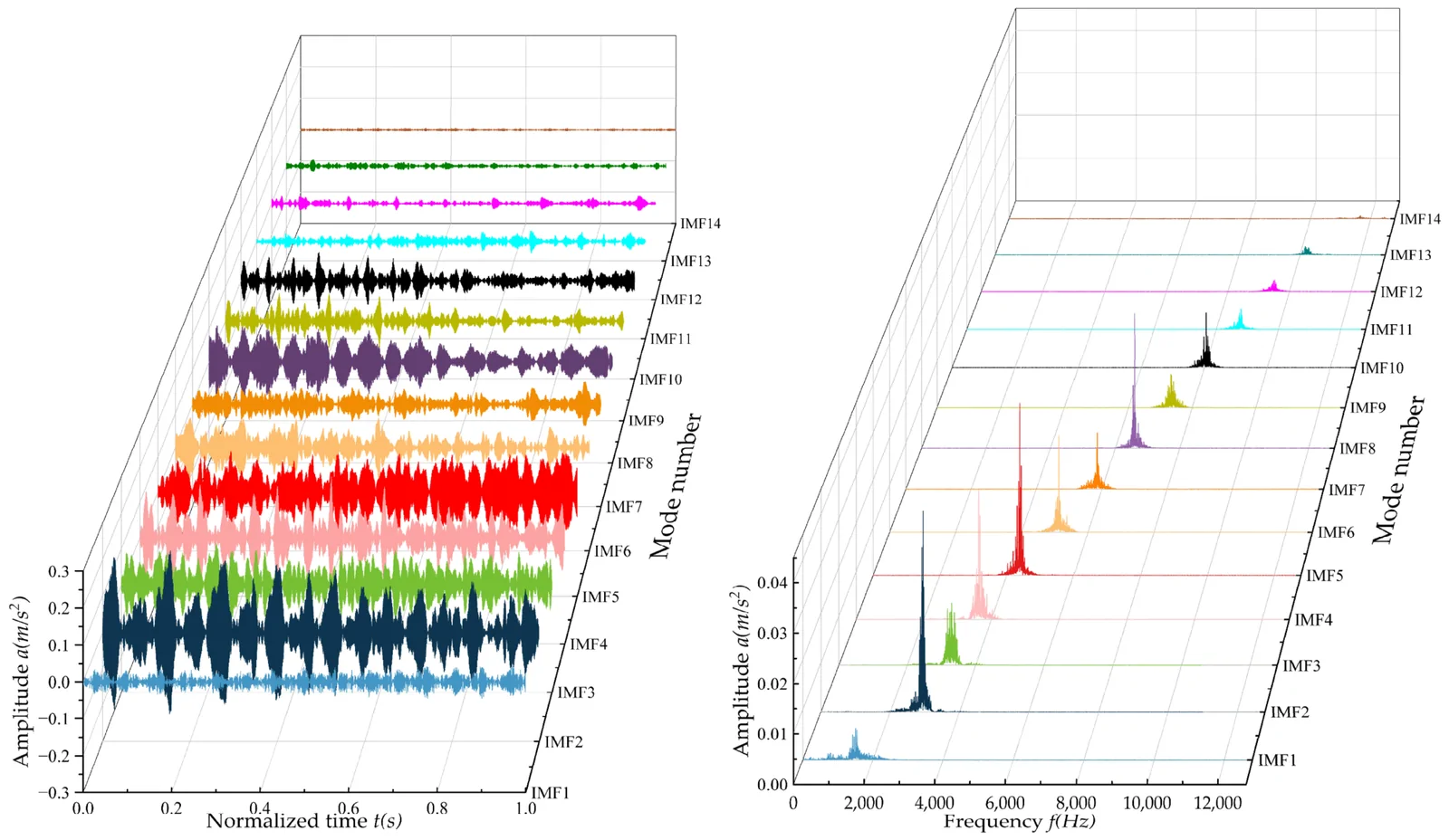
IWHO-VMD results for the RV_Reducer@PG dataset.

**Figure 9:**
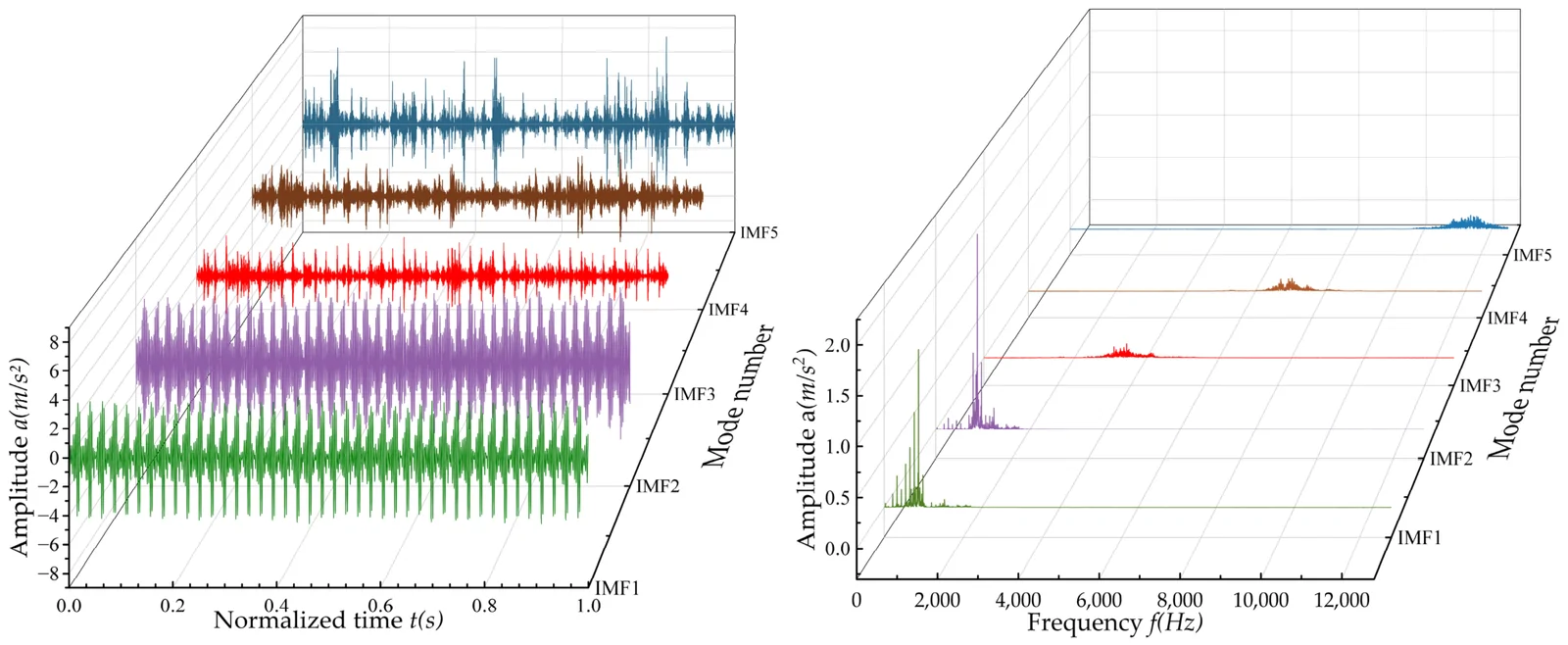
IWHO-VMD results for the RV_Reducer@CS dataset.

Using the optimized parameter combinations obtained in Section 4.1, VMD was applied to representative vibration signals from the three fault datasets. The corresponding decomposition results for RV_Reducer@BB, RV_Reducer@PG, and RV_Reducer@CS are shown in Figure 7, Figure 8 and Figure 9, respectively.

As shown in Figure 7, Figure 8 and Figure 9, the decomposed IMFs exhibit different time-domain waveforms, frequency distributions, impulsive intensities, and energy levels, indicating that fault information is unevenly distributed. Therefore, quantitative mode screening and reconstruction are needed to extract the most fault-sensitive components.

### 4.3. Effective Mode Selection and Signal Reconstruction Results

**Figure 10:**
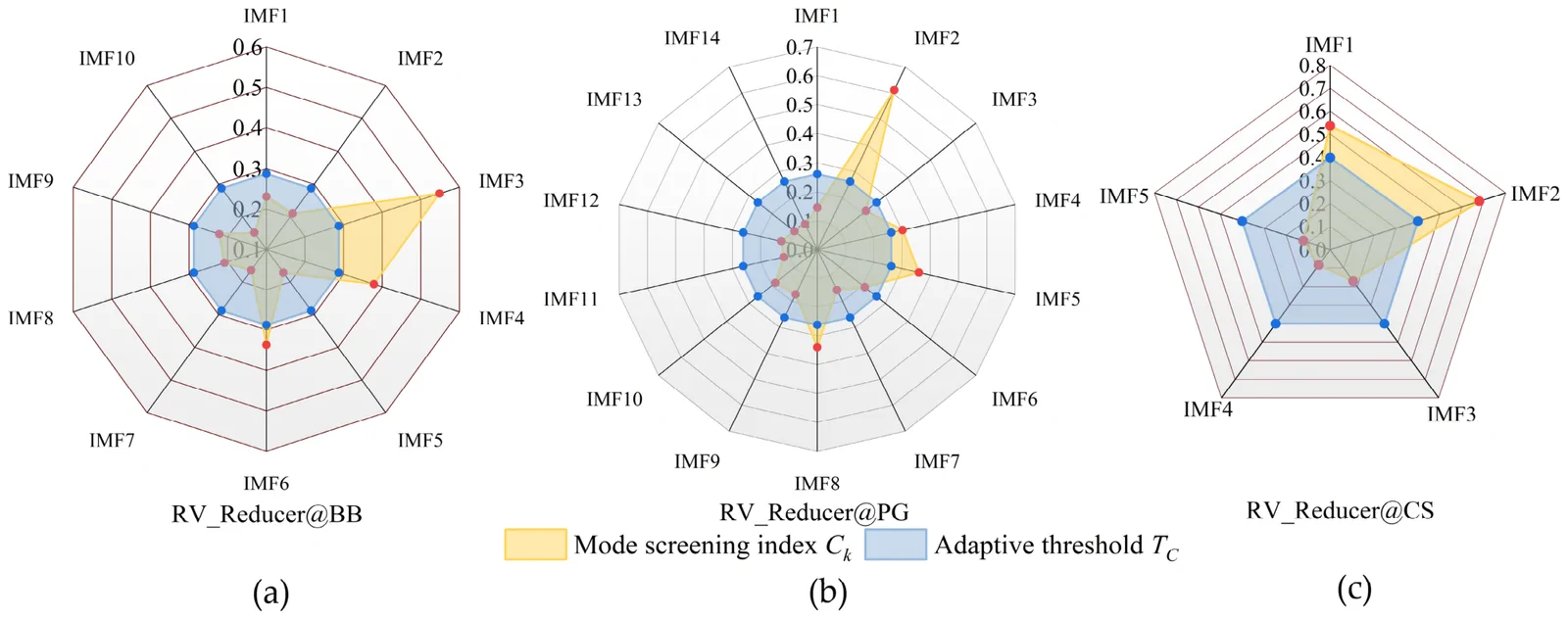
Effective-mode selection results for the three fault datasets: (**a**) RV_Reducer@BB dataset (rolling-element wear); (**b**) RV_Reducer@PG dataset (planetary gear tooth-surface wear); (**c**) RV_Reducer@CS dataset (crankshaft wear).

Based on the effective mode selection method established in Section 3.3, the IMF components obtained from IWHO-VMD are quantitatively evaluated. The mode selection results for the three datasets are presented in Figure 10. For the RV_Reducer@BB dataset, the screening threshold is 0.28754, and IMF3, IMF4, and IMF6 are selected as effective reconstruction modes. For the RV_Reducer@PG dataset, the threshold is 0.26173, and IMF2, IMF4, IMF5, and IMF8 are retained. For the RV_Reducer@CS dataset, the threshold is 0.40032, and IMF1 and IMF2 are selected.

As shown in Figure 10, the retained IMFs have *C_k_* values higher than the corresponding adaptive threshold *T_C_*, indicating greater combined fault-related impulsiveness and energy contribution. These selected modes are then reconstructed to suppress noise-dominated and redundant components while preserving fault-related information, providing a clearer signal basis for subsequent envelope spectrum analysis.

### 4.4. Envelope Spectrum Analysis

Following effective-mode screening and signal reconstruction, Hilbert envelope spectra were obtained for both the original and reconstructed signals of the three datasets. The identified spectral components were interpreted with reference to the theoretical fault frequencies established in Section 3.4.

#### 4.4.1. Rolling-Element Wear

**Figure 11:**
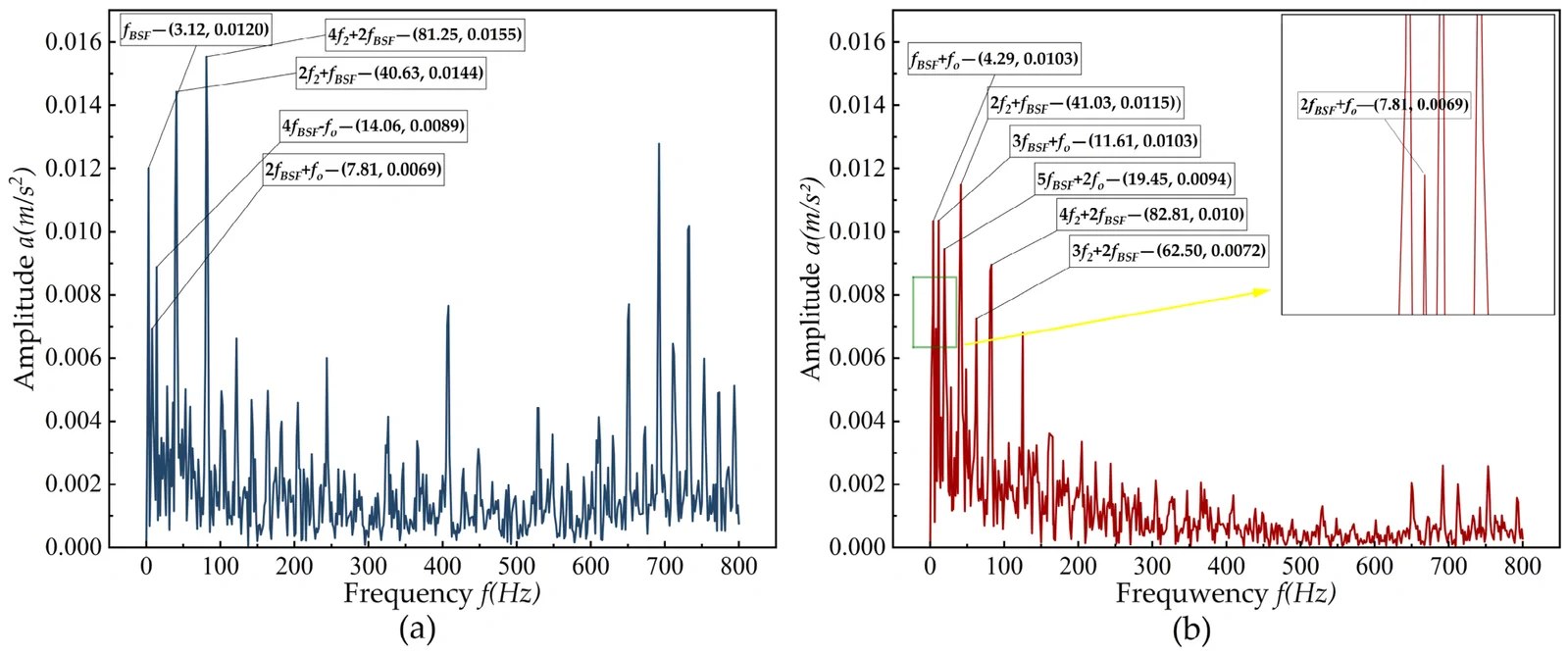
Envelope spectra of the RV_Reducer@BB dataset: (**a**) original signal; (**b**) reconstructed signal.

Envelope spectra of the original and reconstructed signals in the RV_Reducer@BB dataset are compared in Figure 11. According to the structural frequency calculations, the output-disc rotational frequency *f_o_*, rolling-element fault characteristic frequency *f_BSF_*, and crankshaft revolution frequency *f*_2_ are 0.51 Hz, 3.72 Hz, and 18.49 Hz, respectively.

As shown in Figure 11a, the original signal contains several rolling-element-wear-related components, such as *f_BSF_* and 2*f_BSF_* + *f_o_*, but spurious low-frequency peaks and irrelevant high-amplitude components interfere with fault identification. After IWHO-VMD and effective mode reconstruction, Figure 11b shows a lower noise level and clearer spectral structure. Modulation components such as *f_BSF_* + *f_o_*, 2*f_BSF_* + *f_o_*, and 2*f_BSF_* + *f*_2_ are further enhanced, indicating that the reconstructed signal better preserves periodic impacts and modulation information caused by rolling-element wear.

#### 4.4.2. Planetary Gear Tooth-Surface Wear

**Figure 12:**
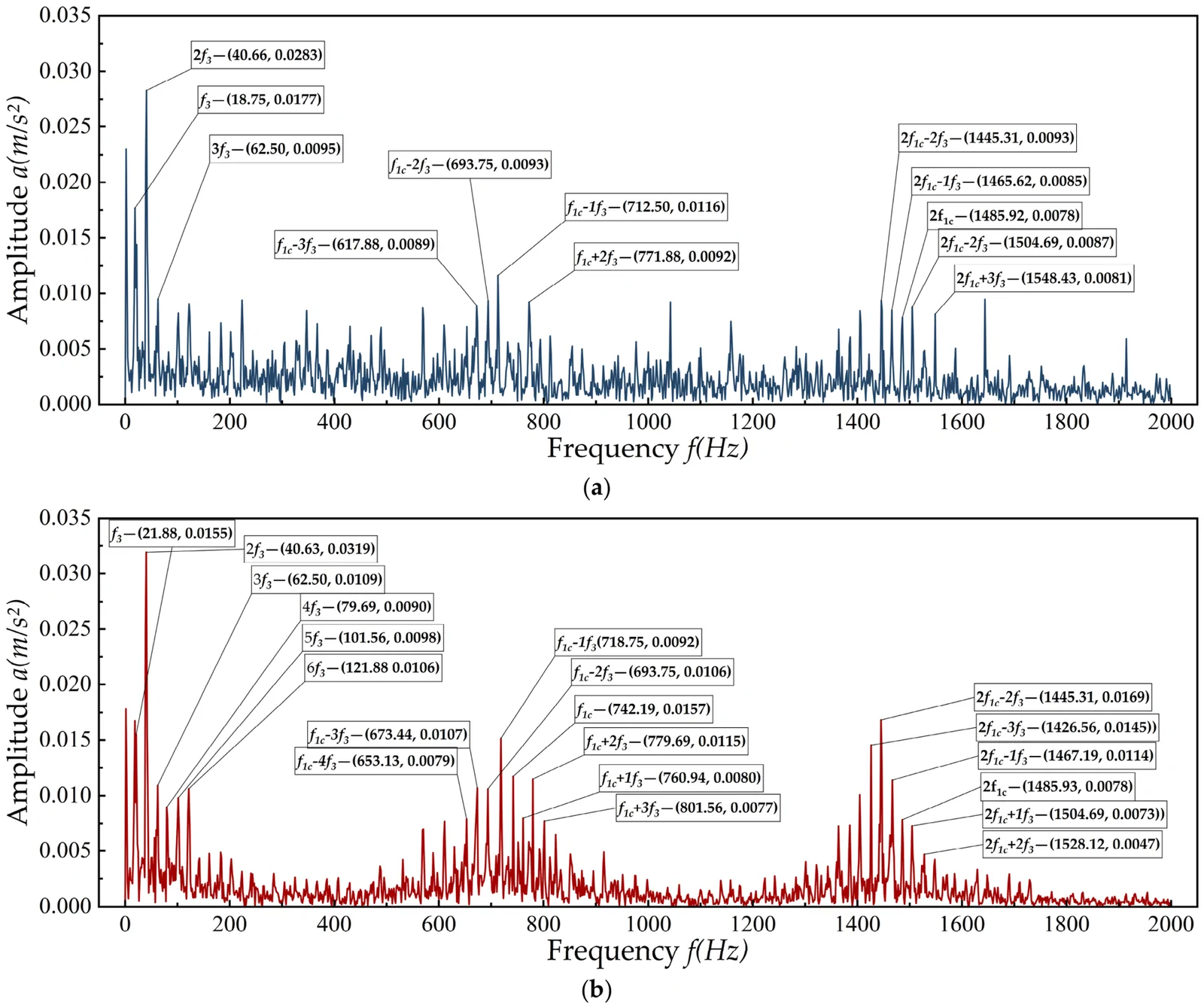
(**a**) Envelope spectrum of the original signal for the RV_Reducer@PG dataset; (**b**) Envelope spectrum of the reconstructed signal for the RV_Reducer@PG dataset.

Envelope spectra of the original and reconstructed signals in the RV_Reducer@PG dataset are compared in Figure 12a,b. According to the structural frequency relationships, the planetary gear self-rotation frequency *f*_3_ is 20.59 Hz, and the sun gear–planetary gear meshing frequency *f*_1*c*_ is 740.8 Hz.

As shown in Figure 12a, the envelope spectrum of the original signal already contains low-frequency fault-related components such as *f*_3_, 2 *f*_3_, and 3 *f*_3_, together with certain modulation responses around *f*_1*c*_ and its harmonics. However, strong background noise and spurious peaks obscure the spectral structure, and the sideband components are not clearly distinguishable. After IWHO-VMD, effective mode selection, and signal reconstruction, Figure 12b exhibits a more complete harmonic chain of *f*_3_, while modulation sidebands such as *f*_1*c*_ ± *lf*_3_ and 2 *f*_1*c*_ ± *lf*_3_ become more readily identifiable. These results indicate that the proposed method effectively preserves the periodic impacts and modulation features induced by planetary gear tooth-surface wear, thereby improving the discriminability of fault-related spectral components.

#### 4.4.3. Crankshaft Wear

**Figure 13:**
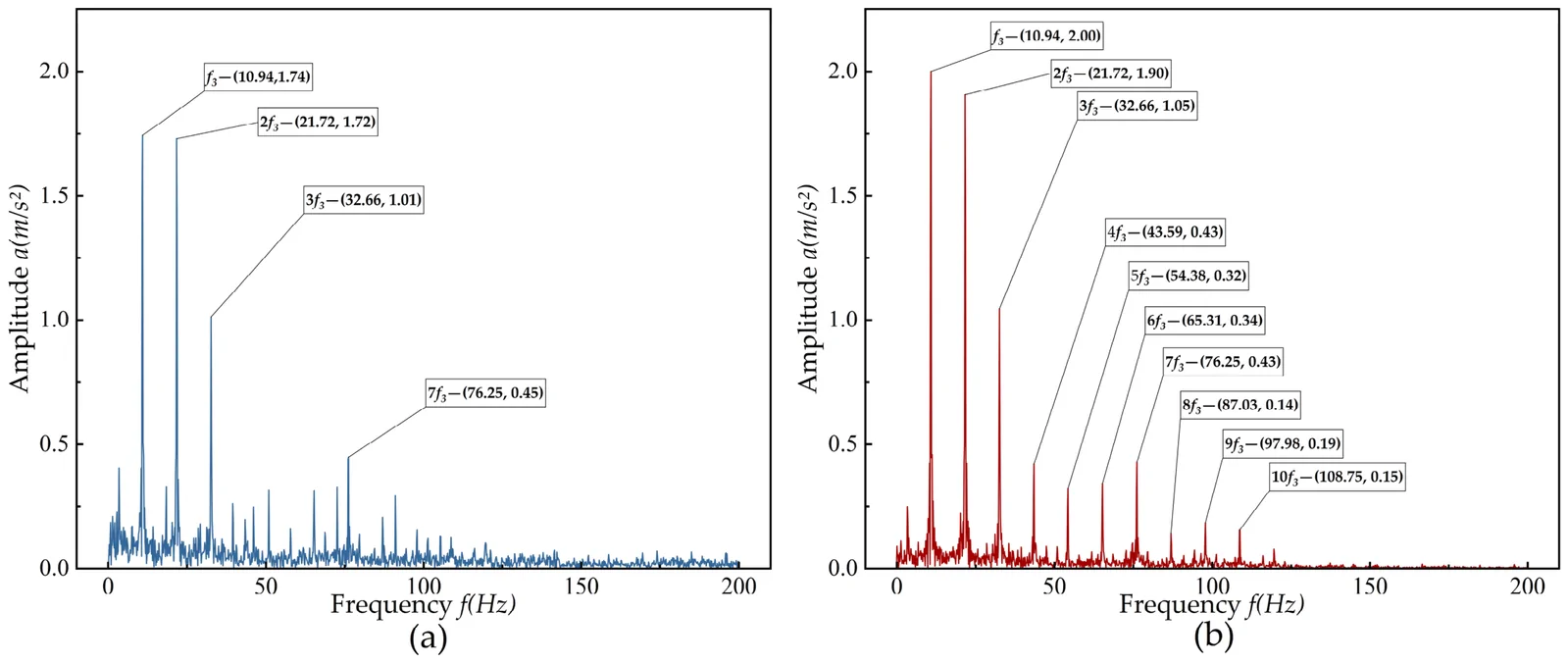
Envelope spectra of the RV_Reducer@CS dataset: (**a**) original signal; (**b**) reconstructed signal.

For the RV_Reducer@CS dataset, the theoretical crankshaft self-rotation frequency *f*_3_ is 11.02 Hz. As shown in Figure 13, components from *f*_3_ to 4*f*_3_ are visible in the original spectrum, whereas the higher-order harmonics remain weak. After decomposition and effective-mode reconstruction, the harmonic structure becomes clearer. The fundamental component is identified at 10.94 Hz, close to the theoretical value, and the higher-order components from 5*f*_3_ to 7*f*_3_ become more pronounced. These results show that the reconstruction procedure improves the visibility of crankshaft-wear-related harmonics.

### 4.5. Characteristic-Frequency Identification Results

To evaluate the identification performance of IWHO-VMD, representative characteristic frequencies of the three fault datasets are summarized in Table 4.

The dataset-level average identification accuracies for RV_Reducer@BB, RV_Reducer@PG, and RV_Reducer@CS are 98.85%, 99.14%, and 98.86%, respectively. The mean of the three dataset-level identification accuracies is 98.95%, while the mean of the corresponding dataset-level relative errors is 1.05%, demonstrating that the proposed method can accurately extract fault-related frequency components for RV reducer fault diagnosis.

### 4.6. Comparative Results

After the proposed method had been evaluated across the three fault datasets, a controlled comparison was conducted on the RV_Reducer@PG dataset using the unified settings defined in Section 3.7. The comparison covered three aspects: parameter-search and convergence performance, VMD quality, and envelope-spectrum-based fault-feature extraction.

#### 4.6.1. Parameter Optimization and Convergence Comparison

**Figure 14:**
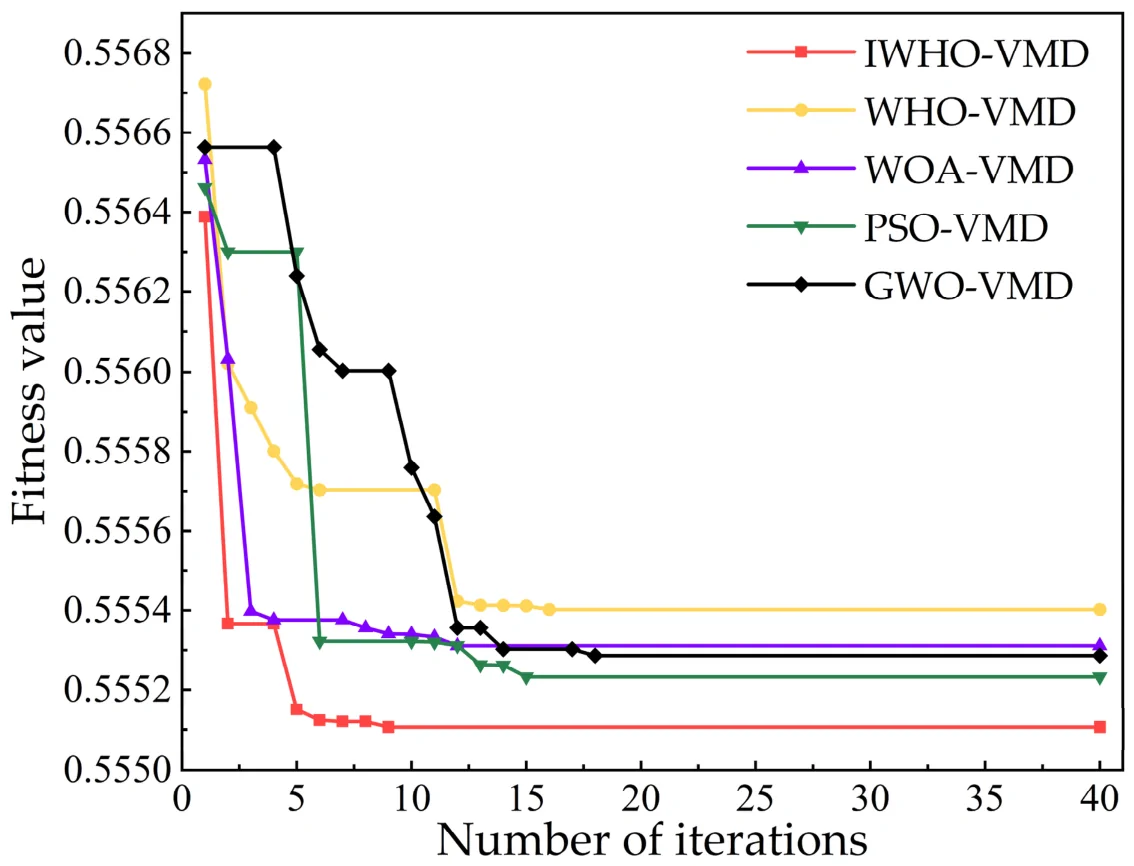
Convergence comparison on the RV_Reducer@PG dataset.

The parameter-optimization results are summarized in Table 5, and the corresponding convergence curves are shown in Figure 14. The different algorithms produced distinct combinations of *K* and α, further confirming the sensitivity of VMD to parameter selection.

As shown in Table 5, IWHO-VMD achieved the lowest mean final fitness value of 0.555106 ± 2.16 × 10^−6^ and required the fewest convergence iterations, with an average of 9.0 ± 0.67 iterations over 10 independent runs. Compared with WHO-VMD, PSO-VMD, WOA-VMD, and GWO-VMD, the mean number of convergence iterations was reduced by 43.8%, 39.6%, 25.0%, and 50.0%, respectively. The pairwise permutation tests based on the Mann–Whitney *U* statistic showed that IWHO-VMD differed significantly from all baseline methods in both the final fitness value and the number of convergence iterations after Holm correction (*p_adj_* < 0.001). These results indicate that the observed improvements were unlikely to be caused solely by random initialization fluctuations.

However, a lower fitness value and fewer convergence iterations do not necessarily ensure lower modal redundancy or better mode separation. The decompositions obtained using the selected parameter combinations were therefore further evaluated using reconstruction error, effective-mode proportion, and mean inter-mode correlation.

#### 4.6.2. Decomposition Quality Comparison

**Figure 15:**
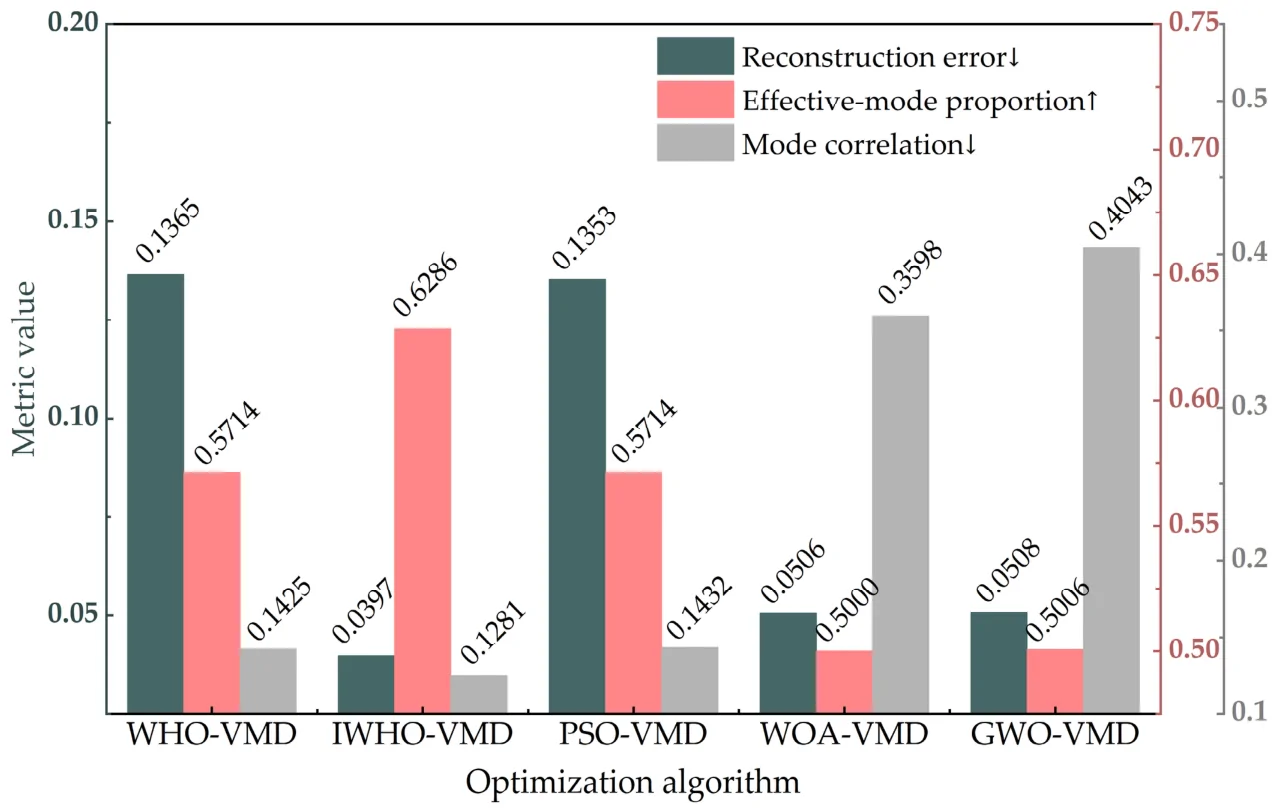
Comparison of reconstruction error, effective-mode proportion, and mean inter-mode correlation among different optimization-based VMD methods on the RV_Reducer@PG dataset.

Figure 15 compares the mean reconstruction error, effective-mode proportion, and mean inter-mode correlation obtained by the five methods over 10 independent runs. Because reconstruction error and effective-mode proportion are related to terms in the composite fitness function, they are used primarily to characterize the resulting decompositions. Mean inter-mode correlation, which is not included directly in the optimization objective, provides a complementary measure of modal dependence and redundancy.

The results demonstrate that IWHO-VMD achieves a more favorable decomposition profile than the benchmark methods. Its mean reconstruction error is reduced to 0.0379, approximately 72% lower than those of WHO-VMD and PSO-VMD, while the effective-mode proportion increases to 0.6286. More importantly, the mean inter-mode correlation remains as low as 0.1281, indicating reduced modal redundancy and enhanced modal independence. Although WOA-VMD and GWO-VMD also yield relatively small reconstruction errors, their lower effective-mode proportions and substantially higher inter-mode correlations suggest that the reduction in reconstruction error does not translate into equally effective mode retention and separation. Overall, IWHO-VMD achieves a superior balance among reconstruction fidelity, effective-information preservation, and modal independence.

**Figure 16:**
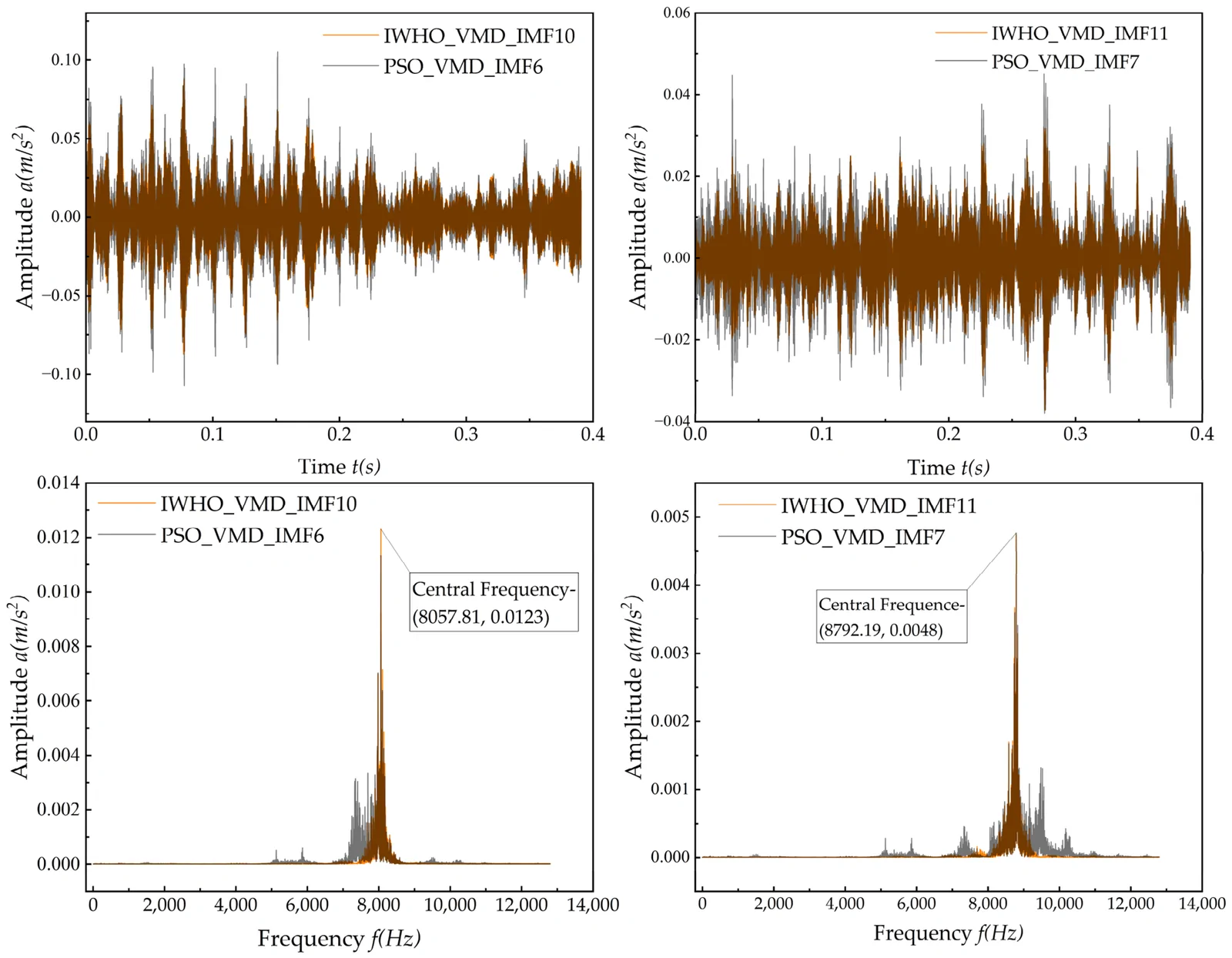
Time- and frequency-domain comparison of representative IMF components obtained by IWHO-VMD and PSO-VMD on the RV_Reducer@PG dataset.

Because mean inter-mode correlation cannot directly characterize local modal overlap, representative IMFs obtained by IWHO-VMD and PSO-VMD within comparable frequency ranges were further compared in both the time and frequency domains, as shown in Figure 16. The time-domain waveforms illustrate the temporal structures of the matched components, while the corresponding spectra show that the IWHO-VMD modes exhibit more concentrated energy around their center frequencies and weaker leakage into adjacent frequency bands. These observations provide complementary visual support for improved mode separation relative to PSO-VMD.

#### 4.6.3. Fault Feature Extraction Comparison

To compare the fault-feature extraction capabilities of the different optimization-based VMD methods, the effective modes obtained by WHO-VMD, PSO-VMD, WOA-VMD, GWO-VMD, and IWHO-VMD were reconstructed, and the corresponding reconstructed signals were analyzed using the Hilbert envelope spectrum. The comparison focused on the prominence of the characteristic peaks, the completeness of the harmonic and modulation-sideband structures, and the detectability of weak fault-related components in the RV_Reducer@PG dataset.

**Figure 17:**
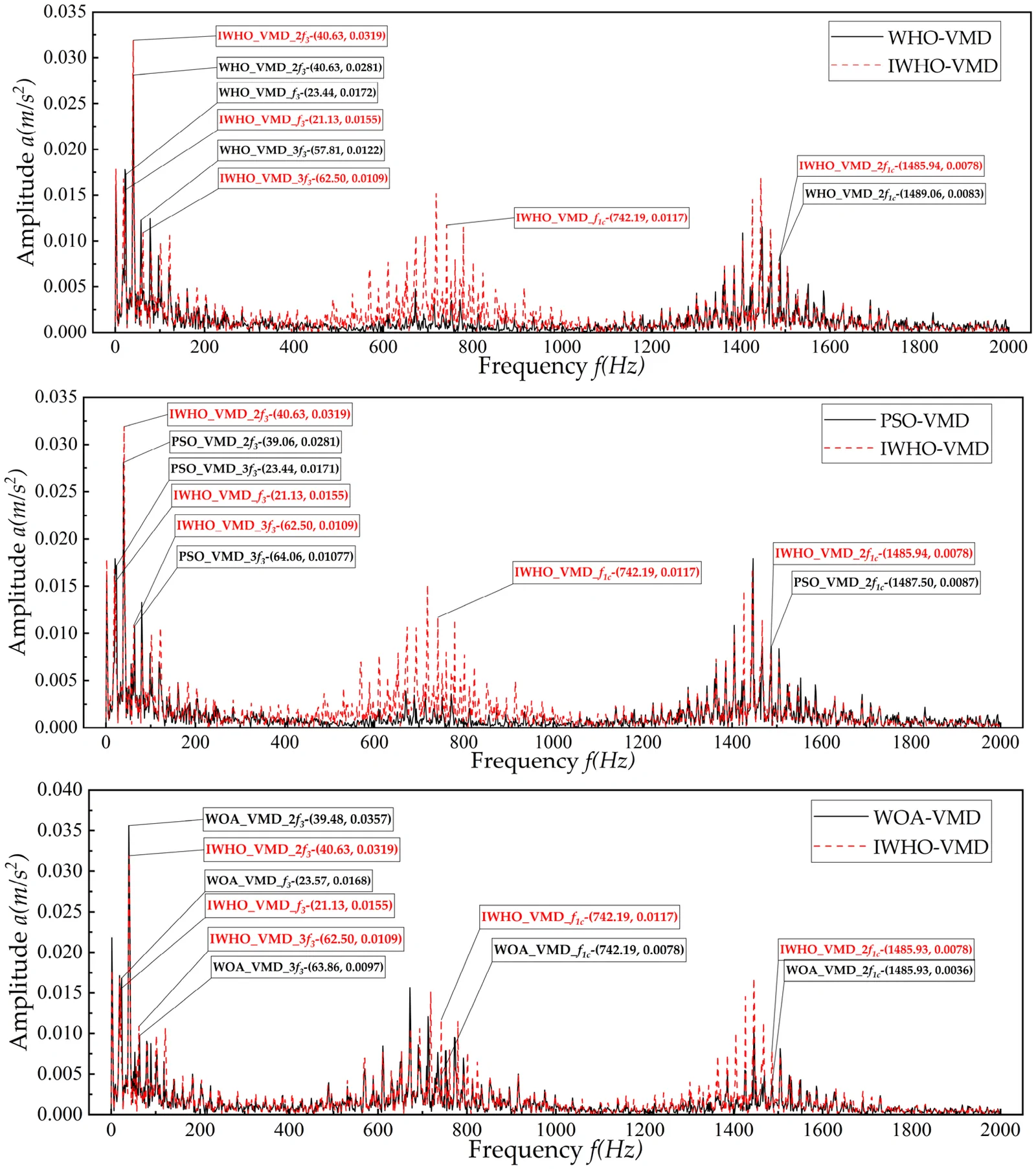

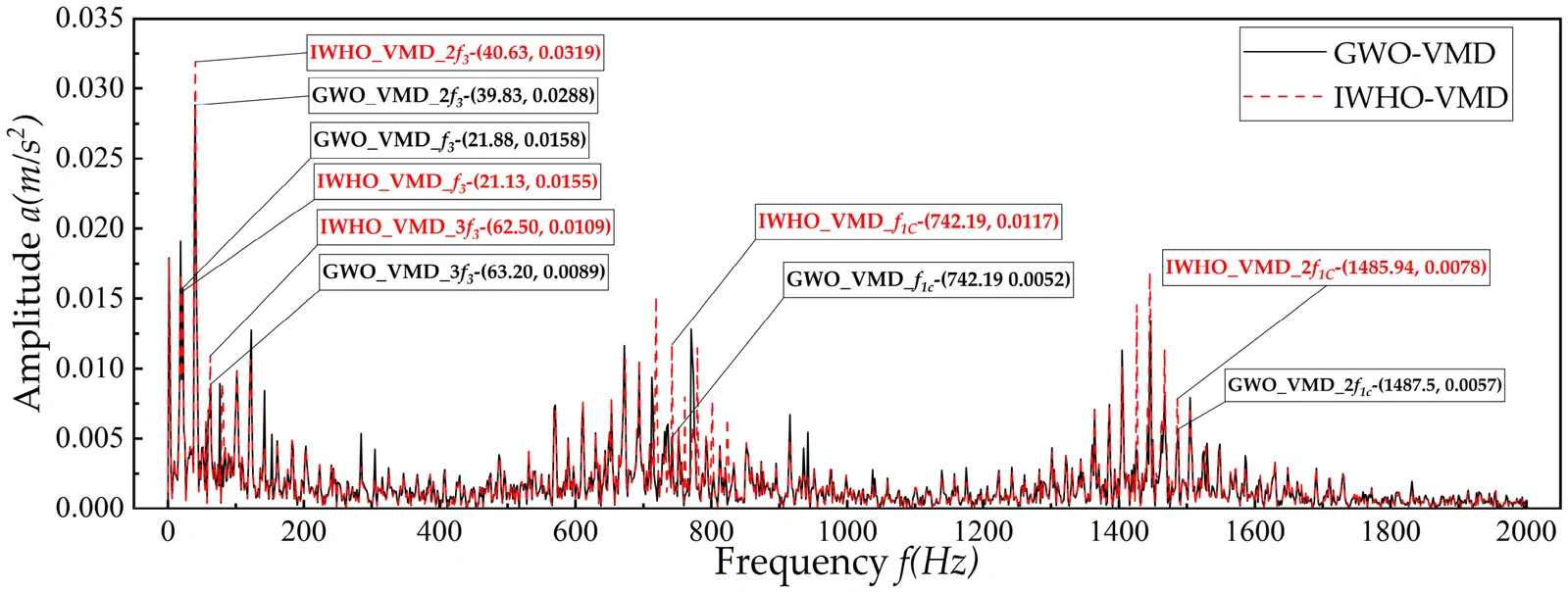
Comparison of envelope-spectrum-based fault feature extraction results among optimization-based VMD methods on the RV_Reducer@PG dataset.

As shown in Figure 17, all compared methods identify several fault-related frequency components, but their envelope spectra differ substantially in peak prominence, spectral completeness, and background interference. Compared with WHO-VMD and PSO-VMD, IWHO-VMD produces more prominent low-frequency components associated with the planetary gear self-rotation frequency and its harmonics. The modulation sidebands around the meshing frequency and its harmonics are also more clearly distinguishable in the IWHO-VMD result. Although WOA-VMD and GWO-VMD identify some of these components, their spectra contain stronger background interference and less complete sideband patterns. These observations indicate that IWHO-VMD preserves the periodic impact and modulation information associated with planetary gear tooth-surface wear more effectively after effective-mode reconstruction.

The quantitative results in Table 6 are consistent with the envelope-spectrum observations. Over 10 independent runs, IWHO-VMD achieved the highest mean PNR (3.1575 ± 0.5312), FPR (0.0772 ± 0.0032), number of identifiable fault features (20.0 ± 1.15), and characteristic-frequency identification accuracy (99.03 ± 0.2335). After Holm correction, the PNR differences between IWHO-VMD and the baseline methods were not statistically significant. The FPR of IWHO-VMD was significantly higher than those of WHO-VMD and PSO-VMD, whereas its differences from WOA-VMD and GWO-VMD were not significant. IWHO-VMD identified significantly more fault-related features than all baseline methods. Its identification accuracy was also significantly higher than those of WHO-VMD, PSO-VMD, and WOA-VMD, but the difference from GWO-VMD did not reach statistical significance (*p_adj_* = 0.0502).

Overall, IWHO-VMD identified more fault-related features and achieved higher identification accuracy than most comparison methods. Although its PNR and identification accuracy were numerically higher than those of GWO-VMD, the differences were not statistically significant.

## 5. Discussion

The results clarify the contribution of IWHO to the parameter-search stage. Tent chaotic initialization is intended to improve initial population coverage, refraction mirror learning helps reduce search stagnation, and the hybrid perturbation combining the Golden Sine Algorithm and MFO strengthens local exploitation near promising solutions. Together, these strategies improve the balance between global exploration and local exploitation, consistent with the lower final fitness values and fewer convergence iterations reported in Figure 14 and Table 5. Because all compared methods use the same subsequent mode-screening and reconstruction procedure, these differences mainly reflect the parameter-search performance of the optimizers.

Fewer convergence iterations, however, do not necessarily imply a shorter overall runtime. A timing check indicated that IWHO-VMD required more computational time than WHO-VMD and PSO-VMD, while remaining comparable to WOA-VMD and GWO-VMD. The additional cost is mainly associated with the learning and perturbation operations within each iteration and the repeated VMD evaluations. The current implementation is therefore more suitable for offline optimization or periodic parameter updates than for real-time re-optimization of every signal window. Further computational acceleration remains necessary for real-time applications.

The effect of parameter optimization is also reflected in the decomposition results. As shown in Figure 15, IWHO-VMD achieves the lowest reconstruction error and mean inter-mode correlation while retaining a relatively high proportion of effective modes. This indicates better reconstruction fidelity and lower redundancy among the decomposed modes, providing a suitable basis for subsequent fault-sensitive mode selection. The sensitivity analysis in Figure 6 further shows that the selected weight combination provides a favorable balance among PNR, FPR, identifiable-feature count, and mean inter-mode correlation. These results support its local robustness within the tested range, without implying theoretical uniqueness or global optimality.

Effective-mode reconstruction further suppresses noise-dominated and redundant components while retaining fault-sensitive IMFs, resulting in clearer harmonic and modulation-sideband structures. On the RV_Reducer@PG dataset, IWHO-VMD identified significantly more fault-related features than all four baseline methods and achieved a mean identification accuracy of 99.03 ± 0.23%. Its accuracy was significantly higher than those of WHO-VMD, PSO-VMD, and WOA-VMD, whereas the difference from GWO-VMD was not significant after Holm correction. These results are consistent with the coordinated contribution of adaptive parameter optimization and effective-mode reconstruction.

The close agreement between the identified and theoretical characteristic frequencies across the rolling-element wear, planetary gear tooth-surface wear, and crankshaft wear datasets indicates potential applicability to different fault components and data sources. However, comparisons with the baseline optimization algorithms were conducted only on the RV_Reducer@PG dataset. The comparative advantage should therefore be interpreted within the present experimental setting.

Several limitations remain. The self-built faults were artificially introduced and may produce more distinct vibration signatures than naturally developing incipient faults. Moreover, the experiments were conducted under a constant load of 12 N⋅m, and sensor placement was not systematically evaluated across multiple positions. The current results therefore mainly demonstrate fault-feature extraction performance under the investigated conditions. Future work should examine naturally evolving faults, variable-load and strong-noise conditions, multi-position measurements, and field-acquired data.

## 6. Conclusions

This study developed an IWHO-VMD framework for fault-feature extraction from RV reducer vibration signals. The framework integrates a two-stage parameter-search strategy, composite fitness evaluation, effective-mode screening, signal reconstruction, and Hilbert envelope-spectrum analysis to improve VMD parameter adaptability and weak fault-feature representation.

First, based on 10 independent runs on the RV_Reducer@PG comparative dataset, IWHO-VMD achieved the lowest mean final fitness value and required the fewest convergence iterations on average. The differences from all baseline methods were statistically significant after Holm correction (*p_adj_* < 0.001), and the mean number of convergence iterations was reduced by 25.0–50.0%. The decomposition-quality comparison further showed that the proposed method reduced reconstruction error and inter-mode redundancy, providing a more reliable basis for subsequent signal reconstruction.

Second, validation on rolling-element wear, planetary gear tooth-surface wear, and crankshaft wear datasets showed that effective-mode reconstruction suppressed noise-dominated and redundant components while preserving fault-related harmonics and modulation sidebands. The dataset-level identification accuracies were 98.85%, 99.14%, and 98.86%, respectively, yielding an overall mean accuracy of 98.95% and a mean relative error of 1.05%. These results demonstrate consistent frequency-identification performance across different fault components and data sources.

Third, on the RV_Reducer@PG dataset, IWHO-VMD achieved mean PNR, FPR, and identification accuracy values of 3.1575, 0.0772, and 99.03%, respectively. It identified significantly more fault-related features than all four baseline methods. Its identification accuracy was significantly higher than those of WHO-VMD, PSO-VMD, and WOA-VMD, whereas the difference from GWO-VMD was not significant after Holm correction.

However, because the self-built rolling-element and planetary gear faults were artificially introduced, the current results do not establish the detection capability of the method for naturally evolving incipient faults. Further validation using naturally evolving degradation data and field-acquired signals under variable-speed, variable-load, strong-noise, and compound-fault conditions is required to assess its practical generalizability.

## Figures and Tables

**Table 1:** Main parameters of the RV20E reducer.

Parameter	Symbol	Value
Reduction ratio	*i_r_*	81
Number of sun gear teeth	*z*_1_	18
Number of planetary gear teeth	*z*_2_	36
Number of cycloidal gear teeth	*z*_3_	39
Number of pin housing teeth	*z*_4_	40
Number of main bearing rolling elements	*N_b_*	34
Main bearing ball diameter/mm	*Db*	7
Main bearing pitch diameter/mm	*Dp*	101.5
Main bearing contact angle/°	θ	40

**Table 2:** Theoretical vibration frequencies of selected RV reducer components.

Component Frequency	Formula
Input speed	*n*
Output-disc rotational frequency	$f_{o}=n/60i_{r}$
Rolling-element fault frequency of the output-end bearing	$f_{BSF}=\frac{D_{p}}{2D_{b}}f_{o}\left[1-{\left(\frac{D_{b}}{D_{p}}\cos\theta \right)}^{2}\right]$
Self-rotation frequency of the input shaft and sun gear	$f_{1}=n/60$
Revolution frequency of the planetary gear and crankshaft	$f_{2}=\frac{z_{1}z_{2}}{\left(z_{3}-z_{4}\right)\left(z_{1}+z_{2}z_{4}\right)}f_{1}$
Self-rotation frequency of the planetary gear and crankshaft	$f_{3}=\frac{z_{1}z_{4}}{\left(z_{3}-z_{4}\right)\left(z_{1}+z_{2}z_{4}\right)}f_{1}$
Meshing frequency between the sun gear and planetary gear	$f_{1c}=\frac{z_{1}z_{2}z_{4}}{z_{1}\left(z_{4}-z_{3}\right)+z_{2}z_{4}}f_{1}$

**Table 3:** Unified parameter settings for the comparative experiments of different optimization algorithms.

Parameter	Setting
Sampling frequency	25.6 kHz
Population size	30
Maximum number of iterations	100
Search range of *K*	[4, 15]
Search range of α	[800, 5000]
VMD parameters	τ = 0, DC= 0, init = 1, tol = 10^−7^
Convergence stability threshold *S_h_*	1 × 10^−5^
Consecutive stability count threshold *S_t_*	20
Number of independent runs	10

where τ denotes the dual ascent step size, DC specifies whether the first mode is constrained to a zero center frequency, init defines the initialization scheme of the mode center frequencies, and tol is the convergence tolerance for the iterative update.

**Table 4:** Identification results of characteristic frequencies for different faults using the IWHO-VMD method.

Dataset	Fault Characteristic Frequency	Theoretical Frequency (Hz)	Identified Frequency (Hz)	Relative Error (%)	Characteristic-Frequency Identification Accuracy (%)	Average Identification Accuracy (%)
RV_Reducer@BB	$f_{BSF}+f_{o}$	4.23	4.29	1.42	98.58	98.85
	$2f_{BSF}+f_{o}$	7.95	7.81	1.76	98.24	
	$3f_{BSF}+f_{o}$	11.67	11.61	0.51	99.49	
	$f_{BSF}+2f_{2}$	40.70	41.03	0.81	99.19	
	$2f_{BSF}+4f_{2}$	81.4	82.81	1.73	98.27	
	$2f_{BSF}+3f_{2}$	62.91	62.50	0.65	99.35	
RV_Reducer@PG	$f_{3}$	20.59	21.13	2.63	97.38	99.14
	2$f_{3}$	41.18	40.63	1.34	98.66	
	$3f_{3}$	61.77	62.50	1.18	98.82	
	$f_{1c}+f_{3}$	761.39	760.94	0.06	99.94	
	$f_{1c}-f_{3}$	720.21	718.75	0.20	99.80	
	$2f_{1c}+f_{3}$	1502.19	1504.69	0.17	99.83	
	$2f_{1c}-f_{3}$	1461.01	1467.19	0.42	99.58	
RV_Reducer@CS	$f_{3}$	11.02	10.94	0.73	99.27	98.86
	2$f_{3}$	22.02	21.72	1.36	98.86	
	$3f_{3}$	33.05	32.66	1.18	98.82	
	$4f_{3}$	44.07	43.59	1.09	98.71	
	$5f_{3}$	55.09	54.38	1.29	98.71	
	$6f_{3}$	66.11	65.31	1.21	98.79	

**Table 5:** Statistical comparison of parameter optimization and convergence performance over 10 independent runs.

Optimization Method	[*K, α* (Mean ± SD)]	Final Best Fitness Value (Mean ± SD)	Convergence Iterations (Mean ± SD)	Relative Reduction by IWHO-VMD (%)
IWHO-VMD	[14, 2188 ± 3.33]	0.555106 ± 2.16 × 10^−6^	9 ± 0.67	-
WHO-VMD	[7, 1102 ± 4.35]	0.555402 ± 3.92 × 10^−6^ *	16 ± 1.41 *	43.8
PSO-VMD	[7, 1086 ± 6.50]	0.555232 ± 5.06 × 10^−6^ *	15 ± 1.37 *	39.6
WOA-VMD	[14, 1025 ± 3.74]	0.555311 ± 2.40 × 10^−6^ *	12 ± 1.76 *	25.0
GWO-VMD	[14, 1089 ± 7.27]	0.555268 ± 7.06 × 10^−6^ *	18 ± 1.70 *	50.0

Note: Values are reported as mean ± standard deviation over 10 independent runs. Lower fitness values and fewer convergence iterations indicate better performance. * indicates a statistically significant difference from IWHO-VMD based on a two-sided permutation test using the Mann–Whitney *U* statistic, with Holm correction (*p_adj_* < 0.001).

**Table 6:** Statistical comparison of envelope-spectrum-based fault feature extraction performance over 10 independent runs.

Optimization Method	PNR (Mean ± SD)	FPR (Mean ± SD)	Number of Identifiable Features (Mean ± SD)	Characteristic-Frequency Identification Accuracy (Mean ± SD, %)
IWHO-VMD	3.1575 ± 0.5312	0.0772 ± 0.0032	20 ± 1.15	99.03 ± 0.2335
WHO-VMD	2.5525 ± 1.1284	0.0680 ± 0.0091 *	14 ± 1.15 *	97.10 ± 0.9475 *
PSO-VMD	2.5254 ± 0.6128	0.0674 ± 0.0039 *	14 ± 1.49 *	97.12 ± 0.3667 *
WOA-VMD	2.6015 ± 1.2138	0.0749 ± 0.0088	18 ± 1.15 *	97.58 ± 0.9718 *
GWO-VMD	2.5420 ± 1.3004	0.0738 ± 0.0096	17 ± 1.76 *	98.30 ± 0.8502

Note: Values are reported as mean ± standard deviation over 10 independent runs. Higher values indicate better fault feature extraction performance. * indicates a statistically significant difference from IWHO-VMD based on a two-sided permutation test using the Mann–Whitney *U* statistic, with Holm correction (*p_adj_* < 0.05).

## Data Availability

The original contributions presented in the study are included in the article; further inquiries can be directed to the corresponding author.

## Abbreviations

The following abbreviations are used in this manuscript:

RV	Rotate Vector
VMD	Variational Mode Decomposition
IWHO-VMD	Improved Wild Horse Optimizer-based Variational Mode Decomposition
WHO-VMD	Wild Horse Optimizer-based Variational Mode Decomposition
WOA-VMD	Whale Optimization Algorithm-based Variational Mode Decomposition
WSO-VMD	White Shark Optimizer-based Variational Mode Decomposition
PSO-VMD	Particle Swarm Optimization-based Variational Mode Decomposition
IMFs	Intrinsic Mode Functions
EWT	Empirical Wavelet Transform
FPR	Fault Peak Ratio
PNR	Peak-to-Noise Ratio
RMS	Root-Mean-Square
SES	Squared Envelope Spectrum
SST	Synchrosqueezing Transform
